# Research progress on the pathogenesis, clinical impact, and traditional Chinese medicine treatment of polycystic ovary syndrome complicated by insulin resistance

**DOI:** 10.3389/fphar.2025.1661806

**Published:** 2025-09-11

**Authors:** Zepu Sun, Bao Jin, Han Han, Zhen Qin, Yuqian Shi, Yuehui Zhang

**Affiliations:** ^1^ Heilongjiang University of Chinese Medicine, Harbin, Heilongjiang, China; ^2^ Key Laboratory and Unit of Infertility in Chinese Medicine, Department of Obstetrics and Gynecology, First Affiliated Hospital, Heilongjiang University of Chinese Medicine, Harbin, Heilongjiang, China; ^3^ Department of Obstetrics and Gynecology, The First Affiliated Hospital of Dalian Medical University, Dalian, Liaoning, China; ^4^ The First Affiliated Hospital of Harbin Medical University, Harbin, Heilongjiang, China

**Keywords:** polycystic ovary syndrome, insulin resistance, traditional chinesemedicine, pathogenesis, signal pathway

## Abstract

**Background:**

Polycystic ovary syndrome (PCOS) is one of the most common reproductive endocrine disorders affecting women of reproductive age. Insulin resistance (IR) is both a hallmark clinical feature and a key contributor to the pathophysiology of PCOS. Currently, metformin, along with other pharmaceuticals and lifestyle modifications, constitutes the primary approach to enhancing IR in PCOS. Despite demonstrating efficacy, some individuals exhibit suboptimal responses, and prolonged usage may lead to gastrointestinal side effects and other constraints. As an important complementary alternative medicine, recent research has highlighted traditional Chinese medicine (TCM) as a valuable adjunctive therapy for ameliorating IR in PCOS. The integration of TCM into the management of PCOS-related IR offers diverse therapeutic avenues, warranting comprehensive categorization and analysis.

**Aim:**

This review systematically summarizes the pathogenesis and TCM interventions of IR in PCOS and its adverse clinical effects on patients at various stages. It primarily focuses on recent research findings, encompassing both animal studies and human studies, regarding the efficacy of TCM in ameliorating PCOS in conjunction with IR over the past 5 years.

**Methods:**

This article collects relevant literature from databases such as PubMed, Web of Science, Embase, and Cochrane Library from the establishment to 2025. The search utilized the following keywords: Polycystic ovary syndrome, Insulin resistance, Polymorphism, Genetic, Epigenomics, Hyperandrogenism, Inflammation, Microbiota, Mitochondria. This review focuses on recent literature published within the last 5 years to maintain the research’s contemporary relevance. Additionally, classical studies are incorporated to uphold the theoretical framework’s integrity.

**Results:**

The current evidence indicates that TCM contributes to the management of PCOS with IR primarily through modulation of gut microbiota equilibrium, suppression of inflammatory reactions (including reduction of inflammatory cytokines), amelioration of hyperandrogenism, and modulation of insulin signaling pathways.

**Conclusion:**

This review examines current research on the treatment of PCOS complicated by IR using TCM. The findings confirm the efficacy of TCM in ameliorating IR. Discrepancies in dosages and treatment durations of TCM compounds and monomers, as well as batch-to-batch variability in TCM quality, may impact treatment efficacy. Additionally, the translation of animal study outcomes to clinical settings remains unvalidated, necessitating further investigation into the synergistic effects of combined TCM and modern medicine approaches. Future efforts should focus on establishing standardized research protocols and quality control measures, enhancing the evidence base for integrated TCM and Western medicine treatments, and facilitating the translation of basic research findings into clinical practice. These steps are crucial for optimizing the role of TCM in managing PCOS-IR.

## 1 Introduction

Polycystic ovary syndrome (PCOS) is one of the endocrine and metabolic disorders mainly characterized by polycystic ovarian changes, hyperandrogenemia, insulin resistance (IR), etc. (Joham et al., 2025). Its prevalence shows significant regional differences, with approximately 6%–10% in the Asian population, 5.3%–7.0% in Europe, and 5.0%–10.0% in the United States (Miazgowski et al., 2021; Wu et al., 2021; Chiaffarino et al., 2022; Kim et al., 2022; Yu et al., 2023; Meng et al., 2025). This difference may be related to racial genetics, lifestyle, and differences in diagnostic criteria (Wolf et al., 2018; Guo et al., 2021). IR and hyperinsulinemia (HI) are important clinical features of patients with PCOS. PCOS patients with varying degrees of IR exhibit more reproductive and endocrine abnormalities (Meirow et al., 1995; Belani et al., 2018; Hao et al., 2024). Currently, in clinical management of PCOS-related IR, treatment methods such as drugs like metformin and lifestyle interventions are commonly used (Teede et al., 2023). Although metformin has a definite effect in controlling symptoms, in clinical application, some patients may experience gastrointestinal adverse reactions (such as diarrhea and nausea), leading to a decrease in patient compliance (Melin et al., 2023; Hofmann et al., 2025; Saadati et al., 2025). Lifestyle interventions are greatly affected by individual behavioral habits and have poor long - term maintenance effects (Arentz et al., 2021; Al Wattar et al., 2022). Therefore, more complementary and alternative treatment methods need to be explored. Traditional Chinese medicine (TCM), as an important complementary and alternative medical approach, has a long history in the treatment of metabolic diseases and gynecological disorders. As early as the Spring and Autumn and Warring States periods (770 BC - 221 BC), the ancient Chinese medical classic “Huangdi Neijing” had recorded the basic understanding of the female menstrual cycle and gynecological diseases, providing a theoretical basis for the later treatment of gynecological diseases and metabolic disorders with TCM (Hammes, 2013; Ma et al., 2021). In recent years, the potential of TCM in improving PCOS and related metabolic and reproductive abnormalities has gradually attracted attention (Chen et al., 2023; Fu L.-W. et al., 2024). Each has its own advantages, providing multiple options for clinical treatment.

While previous research has begun to uncover the link between PCOS and IR, there is a lack of comprehensive descriptions of IR in PCOS within the current literature. This review aims to address this gap by reviewing the multifaceted pathogenesis of IR in PCOS, outlining the specific implications of IR for PCOS patients at various stages, and consolidating recent advancements in traditional Chinese medicines (both botanical drug formulas and their derived metabolites) for ameliorating PCOS-related IR. The objective is to bridge existing research deficiencies, amalgamate the aforementioned evidence, and establish a basis for the development of more targeted prevention and treatment approaches in clinical practice.

## 2 Pathological mechanism of IR in PCOS

### 2.1 Genetic factors involved in the development of IR in PCOS

The development of PCOS with IR is significantly influenced by genetic factors (Luo et al., 2024; Stamou et al., 2024). Gene polymorphism and epigenetics are the specific topics of discussion in this paragraph.

#### 2.1.1 Genetic polymorphisms contributing to the development of IR in PCOS

Gene polymorphism refers to the presence of multiple genetic variations with a frequency exceeding 1% at a specific gene locus within a population (Townes, 1969; Fareed and Afzal, 2013). These variations can impact phenotypic traits such as disease susceptibility and drug response, serving as the foundation of genetic diversity (Ford, 1957; Townes, 1969). In a Finnish cohort, Zouali et al. (1993)initially documented a link between non-insulin-dependent diabetes and the A2 allele of the XbaI polymorphism in the glycogen synthase gene. Multiple receptor gene polymorphisms have been implicated in the pathophysiology of IR, according to subsequent research (Morris, 1997; Kadowaki et al., 2002). Researchers have discovered a growing number of genetic variants linked to the emergence of IR in PCOS in recent years.

People with the rs4784165GG + GT genotype were shown to have a greater risk of IR than those in the TOX3 rs4784165TT group in a study that included 2082 Chinese Han women with PCOS (Tian et al., 2020). Chen F. et al. (2021) used polymerase chain reaction (PCR) and other analytical methods to analyze 616 PCOS patients and 482 healthy women. In the obese subgroup of PCOS patients, they found that the GALNT2 gene rs4846914AA genotype was associated with significantly higher fasting insulin (FINS) and Homeostatic Model Assessment of Insulin Resistance (HOMA-IR) levels than the rs4846914GG or GA genotype in the non-obese subgroup. Nonetheless, the study did not establish a direct association between the GALNT2 gene rs4846914 and rs214430 Single Nucleotide Polymorphism (SNP) variants with PCOS risk. However, these variants were linked to metabolic traits such as IR. Furthermore, independent research has indicated that the rs1801278 polymorphism of the IRS1 gene, while not influencing susceptibility to PCOS, is correlated with IR (Rasool et al., 2022). Specifically, the GA and AA genotypes at rs1801278 of the IRS1 gene were notably linked to heightened HOMA-IR levels (Rasool et al., 2022). Nevertheless, not every genetic variation was linked to a higher incidence of IR in PCOS individuals. While some genetic variants were not linked to the development of IR, others were found to be linked to a lower risk of IR in PCOS individuals. For instance, the THADA rs13429458 polymorphism did not exhibit a difference in insulin resistance, while the DENND1A rs2479106GG and AG genotypes were linked to a lower risk of IR in PCOS individuals (Tian et al., 2020; Dadachanji et al., 2021).

#### 2.1.2 Epigenetic mechanisms in the development of IR in PCOS

Independent of changes in DNA sequences, epigenetics includes the reversible genetic control of gene expression through processes like DNA methylation and non-coding RNA regulation (Guo et al., 2010; Bannister and Kouzarides, 2011; Greenberg and Bourc’his, 2019). These mechanisms play an essential part in the development and progression of IR in PCOS patients (Kang et al., 2015; Abbott and Dumesic, 2021). While methylation at loci 3 and 4 of the INSR gene was associated with HOMA-IR in PCOS patients, a recent case-control study found a significant correlation between HOMA-IR levels and methylation at locus 4 of the AMHRII gene (Zhong et al., 2021). In a familial case-control investigation, Gao et al. (2024) suggested that by increasing TGF-β1 gene expression, decreased methylation of the promoter region of the transforming growth factor-β1 (TGF-β1) gene may improve IR in PCOS. Zhou et al. (2022) used the KGN human granulosa cell line to either knock down FLOT2 using FLOT2-specific siRNAs or overexpress FTO by transfecting it with pcDNA-FTO plasmids. Their research showed that FTO increased the expression of FLOT2 by decreasing m6A methylation levels and enhancing the stability of FLOT2 mRNA, which in turn caused ovarian granulosa cells to induce IR (Zhou et al., 2022).

IR in PCOS is largely caused by non-coding RNAs, including circular RNAs (circRNAs), long-stranded non-coding RNAs (lncRNAs), and microRNAs (miRNAs) (Lin et al., 2018; Mu J. et al., 2021; Mu L. et al., 2021). By attaching to their target Messenger RNA (mRNA) at several regulatory stages, such as transcription, post-transcription, and translation, miRNAs alter the expression of genes (Guo et al., 2010; Kopp and Mendell, 2018). Zhang et al. (2020b) found that 58 miRNAs were differentially expressed in ovarian tissues in PCOS-IR animal models that were fed a diet high in fat and letrozole. These results provide fresh perspectives on possible treatment targets and approaches that involve ovarian miRNAs in the etiology of IR linked to PCOS. Cumulus cells from PCOS patients with IR and those without insulin resistance (NIR) were used in a recent case-control study (Hu et al., 2020). Molecular biology and bioinformatics analysis revealed that the two groups’ cumulus cells expressed 617 genes and 59 known miRNAs differently (Hu et al., 2020). The study specifically demonstrated how the control of the mitogen-activated protein kinase (MAPK) pathway by the miR-612/Rap1b axis contributes to the growth of IR in PCOS (Hu et al., 2020). Additionally, granulosa cells from PCOS patients showed higher expression of miR-133a-3p and suppression of the PI3K/AKT transmission axis in contrast to normal controls, according to Yang X. et al. (2022). Further research showed that miR-133a-3p affects the expression of proteins linked to glucose metabolism and reduces the activity of the PI3K/AKT pathway, which in turn causes ovarian IR (Yang X. et al., 2022). Furthermore, with the goal to shed light on the interaction between HA and IR in PCOS patients and to propose new research directions, Krentowska et al. (2024) identified miR-27a and miR-320 as prospective biomarkers for impaired glucose metabolism in PCOS with HA.

### 2.2 HA mediates the development of IR in PCOS

HA is a complex clinical condition characterized by elevated androgen levels, affecting a majority of individuals with PCOS (Stewart and Edwards, 1994; Azziz et al., 2004; Rosenfield and Ehrmann, 2016). This disorder is both a contributing factor and a clinical manifestation of PCOS. Early research by Burghen et al., in 1980 established a close relationship between IR and HA in PCOS (Burghen et al., 1980). Later research on human intervention provided more evidence in favor of the idea that anti-androgenic actions are essential for reducing IR (Elkind-Hirsch et al., 1993; Moghetti et al., 1996). Current understanding suggests that androgens contribute to the development of IR by directly or indirectly affecting the insulin signaling pathway (Cussen et al., 2025). Excessive androgens can directly influence insulin signaling through androgen receptors, while HA may also indirectly disrupt insulin signaling by promoting inflammation (Ignjatović et al., 2023; Su et al., 2025).

According to recent research, HA can also induce the occurrence and development of IR in PCOS by causing abnormalities in other indirect pathways (Zhai et al., 2020; Yuan et al., 2021; Aflatounian et al., 2022; Misra et al., 2024; Farhadi-Azar et al., 2025). HA reduces the level of Kisspeptin through the action of androgen receptor (AR), thereby disrupting the protective function of Kisspeptin in inhibiting the PERK/eIF2α pathway and calcium overload, leading to endoplasmic reticulum homeostasis and ultimately resulting in the occurrence of IR (Yuan et al., 2021). Zhai et al. (2020) noted that HA downregulates the levels of the circadian clock gene BMAL1 in the liver and adipose tissue, thereby inhibiting the function of the NAMPT/NAD/SIRT1 pathway, reducing the expression of glucose transporter4 (GLUT4), and finally results in the development of IR in an animal model of PCOS. The study by Aflatounian et al. (2022) found that the NAD^+^ level in skeletal muscle tissue mediates HA-induced IR. In addition, recent studies have proposed that prenatal androgen exposure is also one of the important pathophysiological factors for the formation and progression of IR. After constructing an environment of prenatal hyperandrogen exposure by injecting 5 mg of free testosterone into pregnant female rats on the 20th day of pregnancy, it was discovered that the corpulence of their female offspring increased significantly after birth, impaired glucose tolerance occurred at 3 months of age, and it gradually developed into insulin resistance with age (Farhadi-Azar et al., 2025). Interestingly, we found that not all studies seem to propose the theory that androgens are associated with impaired insulin sensitivity. A recent cross-sectional study showed that Dehydroepiandrosterone sulfate (DHEAS) and androstenedione in lean PCOS patients were positively related to insulin sensitivity. This suggests that androgens may protect insulin sensitivity in lean PCOS (Misra et al., 2024). However, the experimental sample was from a single region, and further studies are needed for the extrapolation of the conclusion.

In fact, the pathophysiology of HA in PCOS patients is significantly influenced by IR as well (Cussen et al., 2025; Su et al., 2025). Mechanistically, compensatory HI brought on by IR can increase the ovary’s luteinizing hormone (LH) receptors, making it more receptive to LH and encouraging the release of androgen (Cara and Rosenfield, 1988; Zhang et al., 2000; Rosenfield and Ehrmann, 2016). A key player in androgen biosynthesis, the CYP17-encoded enzyme P450c17α is increased in response to IR and HI, which increases androgen production (Rosenfield and Ehrmann, 2016; Wang et al., 2019; Sanchez-Garrido and Tena-Sempere, 2020; Zhang et al., 2020a). Additionally, HI linked to IR can inhibit the liver’s production of sex hormone-binding globulin (SHBG), increasing free testosterone and androgen biological activity (Deswal et al., 2018). Furthermore, “tissue-selective sensitivity” to insulin in the adrenal gland has been seen in PCOS-IR patients. As a result of IR, HI interacts with the adrenal gland’s insulin receptors, boosting CYP11B1 enzyme activity and subsequently raising the production of 11-oxygenated androgens generated from the adrenal gland (Walzer et al., 2022). The existence of this harmful loop is supported by recent studies. IR and HA in PCOS have a bidirectional pathological link, according to a recent network meta-analysis, and improving insulin sensitivity is essential to breaking this cycle (Xing et al., 2020).

### 2.3 Inflammation mediates the development of IR in PCOS

Actually, PCOS has long been thought to as a low-grade, chronic inflammation (Zhang Q. et al., 2024). The onset and progression of IR in PCOS individuals are significantly influenced by this persistent low-grade inflammation (Deng et al., 2024; Su et al., 2025). Through a case-control research, Kelly et al. hypothesized as early as 2001 that PCOS patients had a chronic low-grade inflammatory state that is inversely connected with insulin sensitivity (Kelly et al., 2001). A later prospective controlled study also revealed a strong correlation between IR and the inflammatory state in PCOS patients (González et al., 2006). Insulin signaling is primarily impacted by inflammation at the cellular and molecular levels via a variety of mechanisms (Hotamisligil et al., 1994; Yu et al., 2022; Olaniyi et al., 2024). First, by preventing the tyrosine phosphorylation of IRS, the inflammatory factor TNF-α reduces cellular sensitivity to insulin by blocking the downstream transmission of insulin signals (Hotamisligil et al., 1994). Through the JAK-STAT, HIF-1, and PI3K-Akt signaling pathways, inflammatory factors drive the development of PCOS-IR in overweight PCOS patients (Yu et al., 2022). Through the NrF2/HIF1-α pathway, hypothalamic inflammation and pyroptosis also contribute to PCOS-IR episodes (Olaniyi et al., 2024). According to a recent study, monocytes (MNC) from PCOS patients with saturated fat activate the NF-κB pathway, causing an overabundance of TNFα, IL-6, and IL-1β to be secreted. These inflammatory molecules then suppress insulin signaling, which results in PCOS-IR (González et al., 2020; Li X. et al., 2024). Therefore, moderately limiting saturated fat consumption can be a useful strategy for PCOS-IR patients to avoid the onset and progression of PCOS-IR.

### 2.4 Abnormal structure and function of gut microbiota contribute to the development of IR in PCOS

The diverse microbial population found in the human gut is referred to as the gut microbiota. In areas including immune system modulation, gastrointestinal mucosal barrier protection, and nutritional absorption and digestion, these microbial populations coexist harmoniously with the human body (Noverr and Huffnagle, 2004; Bäckhed et al., 2005). Thus, preserving the gut microbiota’s homeostasis is crucial to preserving the body’s regular physiological processes. Recent research has revealed that the structure and function of the gut microbiota are typically aberrant in PCOS patients (Morris, 2020; Li P. et al., 2023). Furthermore, some research has revealed that PCOS patients with IR and those without IR have quite different gut microbiotas (He and Li, 2021). As a result, there has been a lot of interest in the connection between the gut microbiota and IR, the main pathogenic characteristic of PCOS.

The pathogenic theory of “gut barrier-endotoxemia-inflammation” was put forth by Tremellen and Pearce (2012) in 2012. According to this view, intestinal mucosa tight junction protein function is initially compromised by gut microbial dysbiosis, which permits lipopolysaccharide (LPS) to pass from the gut into the bloodstream. After entering the bloodstream, LPS attaches itself to receptors on target cells, triggering intracellular signaling cascades that increase the release of cytokines that promote inflammation. In the end, this harms the insulin signaling system, which results in IR (Tremellen and Pearce, 2012). According to further research, *Bacteroides* vulgatus inhibits the production of interleukin-22 (IL-22) by lowering certain bile acids (such glycodeoxycholic acid) that cause the stomach to produce IL-22, which in turn causes PCOS with IR (Qi et al., 2019). According to Yang et al. (2021), there was a notable rise in *Bacteroides* abundance in PCOS patients and PCOS model mice, which was associated by a drop in cholic acid and an increase in farnesol. Insulin resistance results from this because the farnesoid X receptor (FXR) in the ileum is not sufficiently activated. By attaching to the aryl hydrocarbon receptor (AhR) on the surface of type 3 innate lymphoid cells (ILC3s), Aspergillus tubingensis and its secondary metabolite AT-C1 prevent endogenous ligand activation, suppress IL-22 release, and ultimately cause IR (Wu J. et al., 2025). Apart from the previously described microfunctional abnormalities, it has been established that PCOS-IR is intimately linked to macroabnormalities in the ecological structure of the microbiota and their metabolites. In PCOS patients, the degree of IR is positively connected with a decline in Prevotella_9 abundance (Chen J. et al., 2021). Impaired glucose tolerance is linked to an increase in the Firmicutes/Bacteroidetes ratio and a decrease in the gut microbiota’s α-diversity (Yue et al., 2022). The HOMA-IR and the amount of propionic acid in PCOS patients’ feces have a favorable correlation (Dong et al., 2024). But not every type of gut microbiota causes PCOS-IR to develop. By restoring gut microbiota diversity, increasing the abundance of beneficial bacteria (like Bifidobacterium, *Lactobacillus*, and Butyricoccus), decreasing the abundance of harmful bacteria (like *Helicobacter* and *Bacteroides*), and significantly lowering the levels of pro-inflammatory factors (like IL-6 and TNF-α), Bifidobacterium longum subsp. longum BL21 has recently been demonstrated to significantly improve IR in PCOS mice (Dong et al., 2025). Because the gut microbiota is diverse, strains within the same species may affect diseases differently, and future research into the precise pathogenic pathways is necessary.

Emerging research shows microbiota affects neuroendocrine signaling involved in PCOS. The gut microbiota plays a crucial role in regulating neuroendocrine signals via the “gut-brain axis”, contributing to the pathogenesis of PCOS and associated metabolic disorders, including insulin resistance (Babu et al., 2024; Gautam et al., 2024). Microbial metabolites, such as short-chain fatty acids and tryptophan derivatives, can modulate the central nervous system by circulating in the bloodstream (Eepho et al., 2023; Longo et al., 2023; Monroy et al., 2024). These metabolites influence the secretion pattern of gonadotropin-releasing hormone (GnRH) and disrupt the homeostasis of the hypothalamic-pituitary-ovarian axis (Eepho et al., 2023; Longo et al., 2023; Monroy et al., 2024). Consequently, they trigger metabolic dysregulations like energy metabolism perturbations and IR (Eepho et al., 2023; Longo et al., 2023; Monroy et al., 2024). This pathway of the “gut-brain axis” offers a novel perspective on the link between gut microbiota and PCOS with IR.

### 2.5 Mitochondrial dysfunction mediates IR in PCOS

As an emerging area that has attracted much attention in the research on the mechanism of IR in PCOS in recent years, the exploration of mitochondrial dysfunction and its molecular regulatory mechanism provides a new perspective for understanding the pathology of the disease (Zhou et al., 2024). Specifically, mitochondrial gene polymorphisms, abnormal gene expression, and dysfunction form a cascading regulatory network that jointly promotes the occurrence and development of the disease (Deer et al., 2025; Kobayashi et al., 2025).

Firstly, in terms of mitochondrial gene polymorphism, specific polymorphisms in the mtDNA D-loop region may be involved in the occurrence and development of IR in patients with PCOS by influencing the association between body mass index and IR (He et al., 2023). Secondly, at the gene expression level, the reduced expression of nuclear-encoded genes in the mitochondrial oxidative phosphorylation (OXPHOS) system may mediate the occurrence and development of insulin resistance in the skeletal muscle tissue of PCOS patients, and the decreased expression of PGC-1α may be a key mediating factor (Skov et al., 2007). A recent study constructed an animal model of PCOS with IR and intervened with the mitochondrial-targeted antioxidant MitoQ (Ding et al., 2019). It was found that mitochondrial DNA (mtDNA) mutations, reduced ATP production, decreased membrane potential, and excessive reactive oxygen species (ROS) could promote the occurrence and development of IR in PCOS by triggering oxidative stress and abnormal apoptosis (Ding et al., 2019). This indicates that abnormal mitochondrial function also plays an important role in the occurrence and development of PCOS complicated with IR.

However, the relationship between mitochondrial dysfunction and IR is not unidirectional. IR, in turn, can exacerbate mitochondrial damage, forming an intertwined vicious cycle. A recent human study on women with PCOS found that compared with healthy women, the mtDNA content in PCOS patients was significantly reduced, accompanied by more obvious metabolic abnormalities (Rojo et al., 2024). After adjusting for the HOMA index, the differences in mtDNA content and oxidation levels between PCOS patients and healthy women lost statistical significance (Rojo et al., 2024). This suggests that IR may be the main driving factor for mitochondrial abnormalities in PCOS patients. Another study indicated that mitochondrial dysfunction (manifested as mtDNA deletion, decreased metabolic flexibility, etc.) is closely related to the occurrence and development of PCOS with IR and may form a vicious cycle through interaction with obesity and IR (Varhegyi et al., 2024). Human studies have shown that the high platelet reactivity in PCOS patients is closely related to mitochondrial dysfunction (impaired integrity, imbalance of fission - fusion) (Randriamboavonjy et al., 2015). After treating them with metformin, it was found that metformin can improve mitochondrial integrity through AMPKα1 - dependent Drp - 1 regulation, thereby reducing high platelet reactivity, and this effect is only observed in PCOS patients with IR (Randriamboavonjy et al., 2015). This provides a new perspective on the cardiovascular risk and mitochondrial dysfunction in PCOS with IR patients. Animal studies have also confirmed the negative effect of IR on mitochondrial function. IR leads to a decrease in the mtDNA copy number in mouse metaphase II (MII) oocytes, disordered mitochondrial distribution in germinal vesicle (GV) and MII oocytes, and abnormal mitochondrial inner membrane potential (increased in the GV stage and decreased in the MII stage), ultimately resulting in damage to mouse oocytes (Ou et al., 2012).

## 3 Dysregulation of insulin signaling pathways in PCOS involved in the development of IR

**FIGURE 1:**
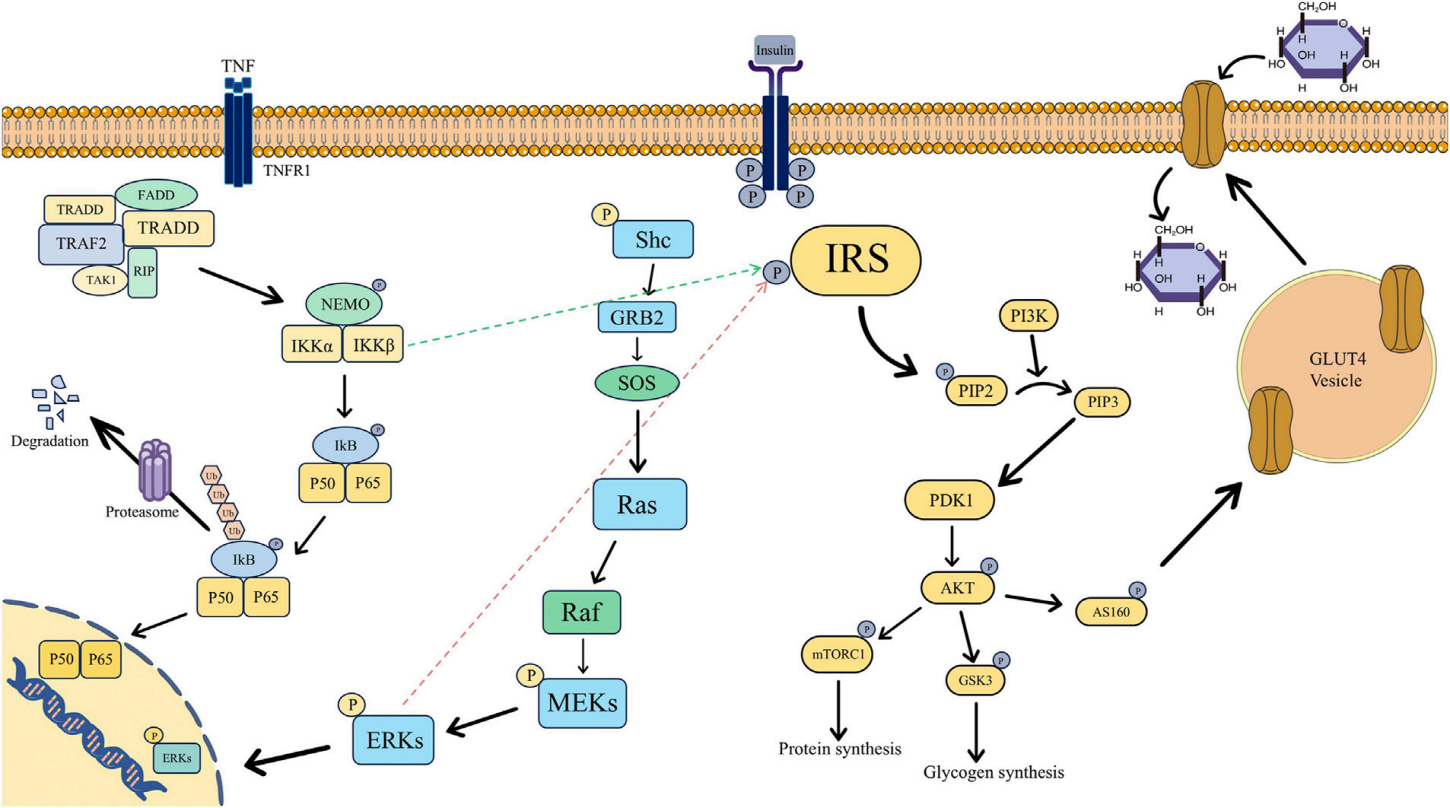
Mechanism diagram of insulin signaling pathways associated with insulin sensitivity in polycystic ovary syndrome, specifically involving the PI3K/AKT, NF-κB, and MAPK pathways. (Abbreviations: P: Phosphorylation, IRS: Insulin Receptor Substrate, PI3K: Phosphoinositide 3-Kinase, PIP2: Phosphatidylinositol 4,5-Bisphosphate, PIP3: Phosphatidylinositol 3,4,5-Trisphosphate, PDK1: 3-Phosphoinositide-Dependent Protein Kinase 1, AKT: Protein Kinase B, mTORC1: mammalian Target of Rapamycin Complex 1, GSK3: Glycogen Synthase Kinase 3, AS160: Akt Substrate of 160 kDa, GLUT4: Glucose Transporter 4, TNF:Tumor Necrosis Factor, TNFR1: Tumor Necrosis Factor Receptor 1, FADD: Fas-Associated Death Domain, TRADD: TNF Receptor-Associated Death Domain, TRAF2: TNF Receptor-Associated Factor 2, TAK1: Transforming Growth Factor-β-Activated Kinase 1, RIP: Receptor-Interacting Protein, NEMO: NF-κB Essential Modulator, IKK: IκB Kinase, IKB: Inhibitor of κB, Ub: Ubiquitin, Shc: Src Homology and Collagen, GRB2: Growth Factor Receptor-Bound Protein 2, SOS: Son of Sevenless, Ras: Rat Sarcoma Viral Oncogene Homolog, Raf: Rapidly Accelerated Fibrosarcoma, MEKs: Mitogen-Activated Protein Kinase Kinases, ERKs: Extracellular Signal-Regulated Kinases).

An essential biological mechanism underlying the onset and progression of PCOS with IR is the aberrant activation or inhibition of the insulin signaling system. The pathogenic process is significantly influenced by the following critical signaling pathways’ malfunction (Figure 1).

### 3.1 Abnormality of the PI3K/AKT signaling pathway mediates the development of IR in PCOS

Under normal circumstances, the tyrosine residues of the β-subunit of the insulin receptor undergo autophosphorylation upon the binding of insulin to its receptor, activating the receptor (Sun et al., 1991). IRS proteins are then recruited and phosphorylated by the active insulin receptor, resulting in their activation (Sun et al., 1991). PI3K is bound to and activated by the active IRS. The transformation of phosphatidylinositol 4,5 bisphosphate (PIP2) on the cell membrane into phosphatidylinositol 3,4,5 trisphosphate (PIP3) is then catalyzed by PI3K. AKT is drawn to the cell membrane by PIP3 (Folli et al., 1992; Hers et al., 2011). Phosphatidylinositol-dependent kinase-1 (PDK1) and mammalian target of rapamycin complex 2 (mTORC2) then work in concert to phosphorylate AKT’s Thr308 and Ser473 sites, which activates the protein (Hers et al., 2011; Huang et al., 2018). Phosphorylation of downstream target proteins is how the activated AKT carries out its actions. To lower blood glucose levels, AKT can also phosphorylate the AS160 protein, which encourages GLUT4 to move from the intracellular compartment to the cell membrane (Xu et al., 2016). This improves cellular glucose uptake and permits blood glucose to enter cells for storage or use. Conversely, GSK-3β is phosphorylated by AKT, which renders it inactive. This relieves the inhibition of GSK-3β, hence facilitating the synthesis of glycogen. Furthermore, AKT phosphorylates the target of rapamycin (mTOR) to stimulate protein synthesis (Cheatham and Kahn, 1995; Saltiel and Kahn, 2001; James et al., 2021).

According to recent studies, PCOS-IR develops and occurs as a result of anomalies at several locations along the insulin signaling pathway. As CPXM1 damages the insulin signaling system, it lowers the expression of IRS-1/2 and INSR while also downregulating the phosphorylation of Akt, a crucial component of the pathway (Pervaz et al., 2023). This results in IR in the ovary and adipose tissue. The primary mechanisms of IR in PCOS, as demonstrated by a recent animal study, are increased serine phosphorylation of IRS-1 and decreased phosphorylation of Akt in the insulin signaling pathway of perigonadal white adipose tissue (pgWAT). This abnormality is closely linked to the inflammatory response mediated by C-C chemokine receptor 5 (CCR5) (Seow et al., 2021). In liver tissue, targeting the reduction of neutrophil extracellular traps (NETs) is the key to improving their negative effect on the phosphorylation of AKT, a downstream signaling molecule of the insulin signaling pathway, and ultimately enhancing insulin sensitivity in the liver tissue (Lin et al., 2025). Src-associated in mitosis, 68 kDa (Sam68) in ovarian granulosa cells lowers the phosphorylation level of IRS-1, which results in inadequate activation of the downstream insulin signaling pathway and, eventually, IR (Vilariño-García et al., 2022).

### 3.2 Abnormality of the NF-κB signaling pathway mediates the development of IR in PCOS

Another important step in fostering the advancement of PCOS with IR is the activation of the classical NF-κB signaling pathway, which is mediated by tumor necrosis factor-α (TNF-α) (Moller, 2000; Shoelson et al., 2006). The NF-κB dimer is inactive in the cytoplasm and firmly binds to Inhibitor of NF-κB alpha (IκBα) under normal physiological conditions. The downstream IκB kinase (IKK) complex is activated in pathological conditions when TNF-α secretion rises sharply and binds to its receptor (TNFR). It then quickly attracts TNF receptor-associated death domain protein (TRADD) and TNF receptor-associated factor 2 (TRAF2) to form a membrane-bound signaling complex (Leong and Karsan, 2000; Oeckinghaus and Ghosh, 2009). When IKK phosphorylates the IκBα protein, it releases the active NF-κB dimer (like p50/p65) and causes the protein to be ubiquitinated and degraded. Following its translocation into the nucleus, NF-κB attaches itself to the κB site in the target gene’s promoter region and starts the transcription of pro-inflammatory proteins like IL-6 and IL-1β (Leong and Karsan, 2000; Oeckinghaus and Ghosh, 2009). These inflammatory factors, in turn, can further stimulate the secretion of TNF-α, forming an inflammatory cascade amplification effect.

From an IR perspective, IKKβ within the NF-κB signaling pathway phosphorylates Ser307 on IRS-1, reducing its capacity for insulin-triggered tyrosine phosphorylation. Consequently, insulin signal transduction is impeded, culminating in IR (Gao et al., 2002; de Alvaro et al., 2004; Liu et al., 2011). Inflammatory mediators like TNF-α, prompted by NF-κB activation, impede PI3K-AKT signaling, hindering GLUT4 translocation to the cell membrane and diminishing peripheral tissue sensitivity to insulin (Hotamisligil et al., 1996; Baker et al., 2011). Prolonged NF-κB activation can exacerbate IR, perturb glucose and lipid metabolism, fostering a detrimental cycle of “TNF-α-NF-κB-inflammation-IR” (Tan et al., 2024).

### 3.3 Abnormality of the MAPK signaling pathway mediates the development of IR in PCOS

Following its binding to target cell receptors, insulin triggers the receptors’ tyrosine kinase activity, which phosphorylates the tyrosine residues of the downstream adapter protein Shc in the pathological process of PCOS with IR (Diamanti-Kandarakis and Dunaif, 2012). Phosphorylated Shc binds to Grb2 and further recruits SOS, which promotes the activation of Ras. Subsequently, the Ras - MAPK cascade reaction composed of Raf - MEK - ERK is activated. Finally, the activated ERK enters the nucleus to phosphorylate transcription factors, thereby regulating the expression of genes related to cell growth and proliferation (de La Fernandes et al., 1999; Corbould et al., 2006; Rajkhowa et al., 2009; Makker et al., 2012; Zhao et al., 2015).

By further activating the pathway, HI in the presence of IR can worsen the development of the disease. After the MAPK pathway is activated, ERK phosphorylates serine sites on IRS-1/2, which prevents IRS from becoming tyrosine phosphorylated and lowers PI3K recruitment (Tanti and Jager, 2009). As a result, the PI3K-AKT pathway’s ability to regulate glucose uptake is impaired, which aids in the development and advancement of IR (Dunaif et al., 2001).

## 4 Negative effects of IR on health in patients with PCOS at different stages

### 4.1 Negative effects of IR on health in adolescent PCOS

IR causes an array of health hazards and worsens clinical characteristics in adolescent PCOS patients. Mechanistically, IR and compensatory HI increase the hypothalamic gonadotropin-releasing hormone (GnRH) pulsatile frequency, which causes the pituitary gland to secrete too much LH (Patel et al., 2003). It exacerbates pre-existing issues such high androgen levels, irregular ovulation, and menstrual cycle disorders by increasing ovarian androgen synthesis and ovulation disorders at the same time (Dunaif, 1997; Venkatesan et al., 2001; Diamanti-Kandarakis and Dunaif, 2012). Furthermore, biochemical hyperandrogenism may exacerbate the clinical manifestations of hyperandrogenism, including acne and hirsutism (Taketani and Mizuno, 1990; Stanczyk, 2006; Guo et al., 2023). As early as 2001, a clinical study demonstrated that most patients’ normal menstrual cycle and the hypothalamic-pituitary-ovarian axis function could be successfully restored, androgen levels could be decreased, after improving insulin sensitivity in white adolescent girls with PCOS with metformin and a high-protein, low-carb diet (Glueck et al., 2001). A retrospective observational study of adolescents with PCOS then examined metabolic markers of several menstruation types, such as oligomenorrhea (OM), secondary amenorrhea (SA), and primary amenorrhea (PA) (Javed et al., 2015). It was discovered that the PA group had a considerably higher HOMA-IR value and fasting insulin level than the SA and OM groups, and that the PA and SA groups had a significantly greater prevalence of metabolic syndrome than the OM group (Javed et al., 2015). This implies that IR is linked to a higher metabolic risk and may be particularly noticeable in teenage PCOS patients who have severe irregularities in their menstrual cycles, such amenorrhea. According to a recent meta-analysis and systematic review, the incidence of amenorrhea in adolescents with PCOS increases with body mass index (BMI) (Zuchelo et al., 2024). This indirectly reflects the correlation between abnormal glucose and lipid metabolism and abnormal menstrual cycles in adolescent PCOS patients.

IR is intimately linked to obesity and poor glucose metabolism in adolescents with PCOS (Bayramoğlu et al., 2021; Gupta et al., 2022; Fu et al., 2023). In adolescents with PCOS, elevated blood glucose and lipid levels are also strongly linked to the onset and progression of a number of metabolic disorders (Huang et al., 2010). Adolescent females with PCOS had higher triglyceride, cholesterol, and IR levels than do those without the condition (Patel-Sanchez et al., 2023). The incidence of non-alcoholic liver disease is greater in adolescents with PCOS who go on to develop diabetes (Patel-Sanchez et al., 2023). This indicates that IR is one of the major pathogenic causes for non-alcoholic liver disease in adolescents with PCOS, in addition to being intimately linked to aberrant glucose and lipid metabolism in these patients. Furthermore, a cross-sectional investigation of obese teenage PCOS patients revealed a strong correlation between fasting IR and the pancreatic fat fraction in teenage girls, which is unrelated to PCOS (Ware et al., 2022). Moreover, IR is significantly linked to poorer sleep quality (including sleep-disordered breathing and decreased sleep efficiency) in obese teenage PCOS patients, indicating that doctors should focus on the holistic care of both (Simon et al., 2020).

However, the psychological problems caused by metabolic disorders are also worthy of attention. Teenagers and young women with PCOS have higher levels of anxiety and sadness than those without the condition, according to a recent systematic review and meta-analysis (Nasiri-Amiri et al., 2023). The causes of negative psychology, including depression and anxiety, in adolescents with PCOS were then investigated in a number of clinical studies that used assessment techniques like questionnaire scale evaluation, group comparative analysis, and correlation analysis. These studies suggested that the presence of negative emotions in adolescents with PCOS may be connected to clinical symptoms like obesity, hirsutism, and irregular menstrual cycles linked to IR (Kara et al., 2022; Saei Ghare Naz et al., 2023). Therefore, the mental health of adolescents with PCOS-IR should be taken into consideration when treating their organic disorders in the future.

### 4.2 Negative effects of IR on the health of women of reproductive age with PCOS

In women with PCOS who are of reproductive age, IR can lead to both metabolic problems and malfunction of the reproductive system (Vatier et al., 2022). This disorder includes infertility, which is defined by anovulatory infertility and aberrant oocyte development, as well as pregnancy problems, such as decreased live birth rates and an increased chance of threatening miscarriage (Dumesic and Abbott, 2008; Sakumoto et al., 2010; Vatier et al., 2022).

On the one hand, IR can cause infertility in women of reproductive age with PCOS through multiple mechanisms. Heightened expression of miR-181d-5p in PCOS patients compared to healthy individuals has been observed (Sang et al., 2024). This upregulation of miR-181d-5p can impede granulosa cell proliferation by inhibiting SIRT1 activity, thereby compromising oocyte quality in PCOS-IR individuals (Sang et al., 2024). In terms of ovulation, IR stimulates the production of androgens (such as testosterone) by inducing compensatory hyperinsulinemia and acting synergistically with the elevated LH level on hypersensitive ovarian theca cells (Graham and Selgrade, 2017). The elevated testosterone induces the synthesis of LH in the pituitary gland and inhibits the function of FSH. Meanwhile, it increases the sensitivity of follicles to LH and causes premature luteinization, ultimately impairing ovulation ability (Graham and Selgrade, 2017). Furthermore, IR can reduce the sensitivity of the ovaries to gonadotropins in PCOS patients, obstructing the follicle development and ovulation processes and thus leading to ovulation disorders (Ezeh et al., 2021; Li Y. et al., 2023).

On the other hand, in women of reproductive age with PCOS, IR may lower the live birth rate and raise the frequency of pregnancy problems. By blocking the PI3K/AKT pathway and inhibiting glycolytic enzymes such as HK2 and PKM2, IR prevents embryo implantation by upsetting endometrial balance and energy supply (Zhang et al., 2024d). Patients with PCOS are therefore more likely to experience miscarriage and unsuccessful embryo implantation. Additionally, IR increases the endometrial inflammatory milieu by promoting the activation of natural killer (NK) cells and skewing macrophages toward the M2 anti-inflammatory phenotype (Liu S. et al., 2021). This chain reaction further hinders the implantation of embryos and jeopardizes the continuation of pregnancy. IR and the rate of prenatal abortions in PCOS are also tightly related. According to an examination of a retrospective cohort study, IR plays a significant role in early abortion in PCOS patients’ first embryo transfer cycle (Chen et al., 2022). According to another study, insulin resistance is linked to a higher risk of preterm delivery in PCOS patients, and PCOS is an independent risk factor for late abortion (Jie et al., 2022). Nevertheless, other research has also demonstrated that IR’s detrimental impact on the late pregnancy outcomes of induced pregnant women is not specific to PCOS patients (Yang T. et al., 2022). By interfering with endometrial homeostasis and energy availability, IR mechanistically reduces pregnancy stability (Zhang et al., 2024d). Additionally, IR can cause and exacerbate endometrial chronic inflammation, increasing the chance of miscarriage in PCOS patients (Liu S. et al., 2021). Additionally, the combined effects of IR and HA cause abnormal mitochondrial shape and function in the placenta and gravid uterus. By blocking the Nrf2/GPX4 pathway, this combination also upsets the balance between oxidation and antioxidation, which leads to unusual cell death pathways like ferroptosis and miscarriage (Hu et al., 2019; Zhang et al., 2019; Zhang Y. et al., 2020). The final result of the multi-link damage described above is a decrease in the live birth rate. Research has indicated that in patients with PCOS, IR is a risk factor in and of itself for the lower live birth rate following frozen-thawed embryo transfer (Jiang et al., 2021). According to another clinical investigation, pregnant women with metabolic syndrome are more likely to develop gestational diabetes and give birth to babies who are macrosomic, and metabolic syndrome has a detrimental effect on the clinical pregnancy rate and live birth rate in obese PCOS patients (Arya et al., 2021). These results highlight the negative effects of metabolic abnormalities, like IR, on the live birth rates and reproductive outcomes of women with PCOS who are of reproductive age.

**FIGURE 2:**
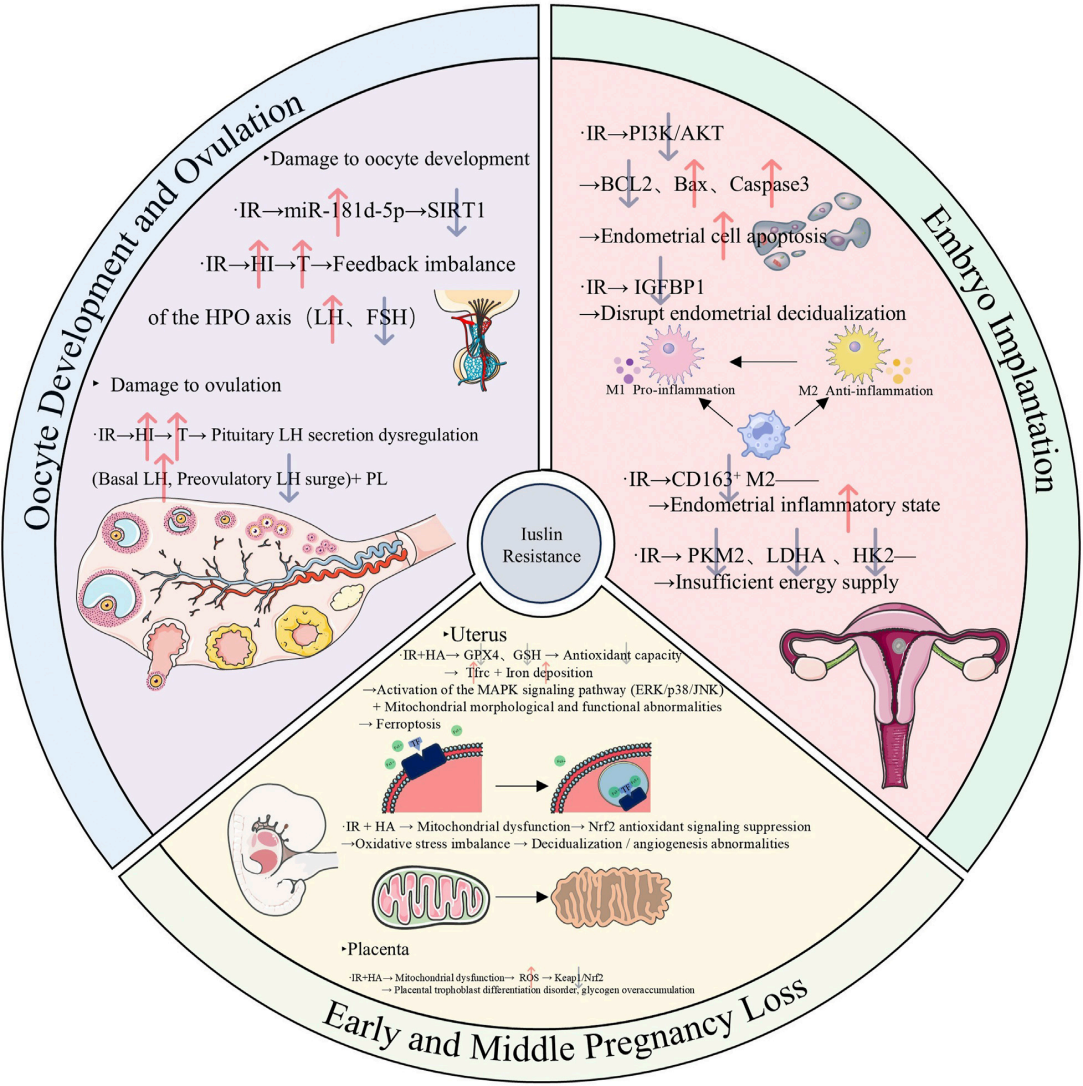
Mechanism diagram illustrating the mechanisms underlying the adverse effects of insulin resistance on the reproductive system in reproductive-aged women with polycystic ovary syndrome, specifically involving oocyte development, ovulation, embryo implantation, and early and middle pregnancy loss. (Abbreviations: IR: insulin resistance, miR-181d-5p: microRNA-181d-5p, SIRT1: sirtuin 1, HI: hyperinsulinemia, T: testosterone, LH: luteinizing hormone, FSH: Follicle-stimulating hormone, BCL2: B-cell lymphoma 2, Bax: Bcl-2-associated X protein, IGFBP1: Insulin-like Growth Factor Binding Protein 1, CD163: Cluster of Differentiation 163, PKM2: Pyruvate Kinase M2,LDHA: Lactate Dehydrogenase A, HK2: Hexokinase 2, GPX4: Glutathione Peroxidase 4, GSH: Glutathione, Tfrc: Transferrin Receptor, Nrf2: Nuclear Factor Erythroid 2-Related Factor 2, ROS: Reactive Oxygen Species, Keap: Kelch-Like ECH-Associated Protein 1).

As mentioned above, IR affects oocyte development, ovulation function, and pregnancy outcomes in women of reproductive age with PCOS through the aforementioned multiple mechanisms, ultimately leading to infertility or increasing the risk of miscarriage. The various regulatory mechanisms discussed above are summarized in (Figure 2).

### 4.3 Negative effects of IR on the health of postmenopausal women with PCOS

In patients with PCOS, IR is not only one of the core pathogenic factors in the pre-menopausal period but also continuously affects the health status after menopause, significantly increasing the risk of various long - term complications (Lambrinoudaki, 2011; Xu and Qiao, 2022). In fact, there are few studies on the multiple disease states of post - menopausal PCOS patients caused by single insulin resistance, and more studies focus on the synergistic effect of IR and other factors such as HA. The core mechanism of the synergistic effect between IR and HA lies in the vicious cycle of their interaction.

IR induced by HA worsens postmenopausal PCOS in rat models, resulting in significant abnormalities in glucose and lipid metabolism as well as cardiovascular and renal damage, including hypertension, decreased glomerular filtration rate, proteinuria, and tubular injury (Dalmasso et al., 2016). The negative effects of IR on postmenopausal cardiac health and glucose-lipid metabolism were highlighted by a recent study that used a postmenopausal PCOS rat model to show that treatment with a GLP-1 receptor agonist (liraglutide) effectively mitigated multiple cardiometabolic risk factors and improved IR (Torres Fernandez et al., 2019). More significantly, compared to people without PCOS, those with PCOS appear to be at a higher risk of developing cancer. With evidently aberrant glucose and lipid metabolism, postmenopausal PCOS patients have a larger chance of developing certain cancers than premenopausal PCOS patients (Meczekalski et al., 2020; Frandsen et al., 2023; Frandsen et al., 2024). The mechanism behind this could be linked to pathological diseases such adipokine imbalance and chronic inflammation brought on by IR alone or in conjunction with hyperandrogenism (Esposito et al., 2014; Slopien et al., 2018; Elibol et al., 2025).

## 5 TCM in the treatment of IR in PCOS

In the realm of complementary and alternative medicine, TCM is increasingly recognized for its significant role in treatment (Dubey and Kumar, 2025). Studies have highlighted the medicinal value of TCM, including compounds and monomers, in enhancing the management of PCOS and regulating endocrine and metabolic functions (Chen et al., 2023; Fu L.-W. et al., 2024). Research has shown promising outcomes in utilizing TCM to address PCOS, particularly in cases complicated by IR. The following paragraphs summarize the cutting-edge studies (covering animal and human studies) of TCM in improving PCOS with IR in recent years (especially in the past 5 years).

### 5.1 Application of TCM botanical drug products alone to improve IR in PCOS

#### 5.1.1 Evidence from animal studies

##### 5.1.1.1 TCM botanical drug formulas

The primary therapeutic modality in TCM clinical practice involves the utilization of compound formulas comprising diverse Chinese botanical drugs, known as “Fufang” in Chinese. Rooted in the holistic principles and the practice of syndrome differentiation and treatment within TCM, these compound formulas exhibit the capacity to ameliorate IR in PCOS through a multifaceted approach, leveraging the synergistic actions of numerous metabolites across various targets and pathways.

**FIGURE 3:**
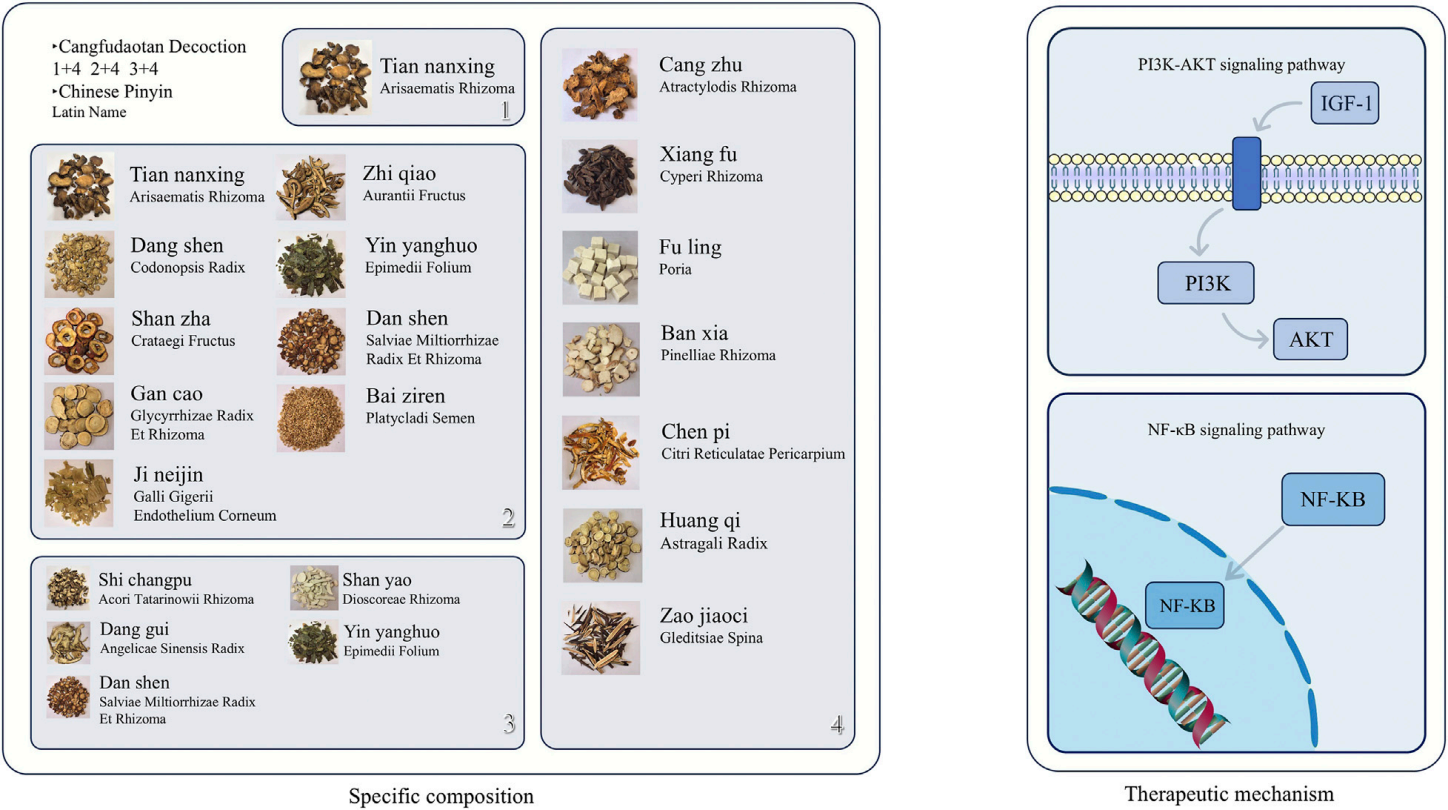
Schematic diagram of the specific composition of Cangfu Daotan Decoction (including Chinese herbal medicine images, Chinese pinyin, and Latin botanical names) and its mechanism of action in improving insulin resistance in polycystic ovary syndrome. The above Chinese Pinyin and Latin names are all sourced from the Chinese Pharmacopoeia (2020 Edition). The specific website of the Chinese Pharmacopoeia: https://ydz.chp.org.cn/#/.

Cangfu Daotan Decoction (CFD) is a classic prescription in TCM, mainly composed of Atractylodes lancea, Cyperus rotundus, and Citrus reticulata. Animal experiments have shown that the basic formula of CFD or its modified formulas can effectively improve PCOS with IR (Figure 3). Wang et al. (2020a) observed the mechanism of action of CFD on PCOS-IR rats. The study found that CFD improves IR by activating the IGF-1-PI3K/Akt pathway (Wang et al., 2020b). Specifically, it upregulates the expression of IGF-1 and promotes the phosphorylation of PI3K and Akt, thereby enhancing insulin signal transduction and ultimately reducing the HOMA-IR value. Jiang et al. (2022) treated a PCOS-IR rat model with CFD or metformin and found that CFD can effectively improve insulin resistance in PCOS-IR model rats, and its efficacy is similar to that of metformin. In addition, the levels of pro-inflammatory factors (such as TNF-α and IL-1) in the serum of rats treated with CFD were significantly reduced. Mechanistically, it is speculated that CFD can effectively alleviate the negative effects of inflammation on the insulin signaling pathway. Liu et al. (2022)reported that treating a PCOS-IR rat model established by letrozole combined with a high-fat diet with Modified CFD can regulate the NF-κB/LCN-2 inflammatory signaling pathway, thereby inhibiting the release of inflammatory factors and upregulating insulin signal-related genes, and ultimately improving the insulin sensitivity of PCOS-IR rats.

**FIGURE 4:**
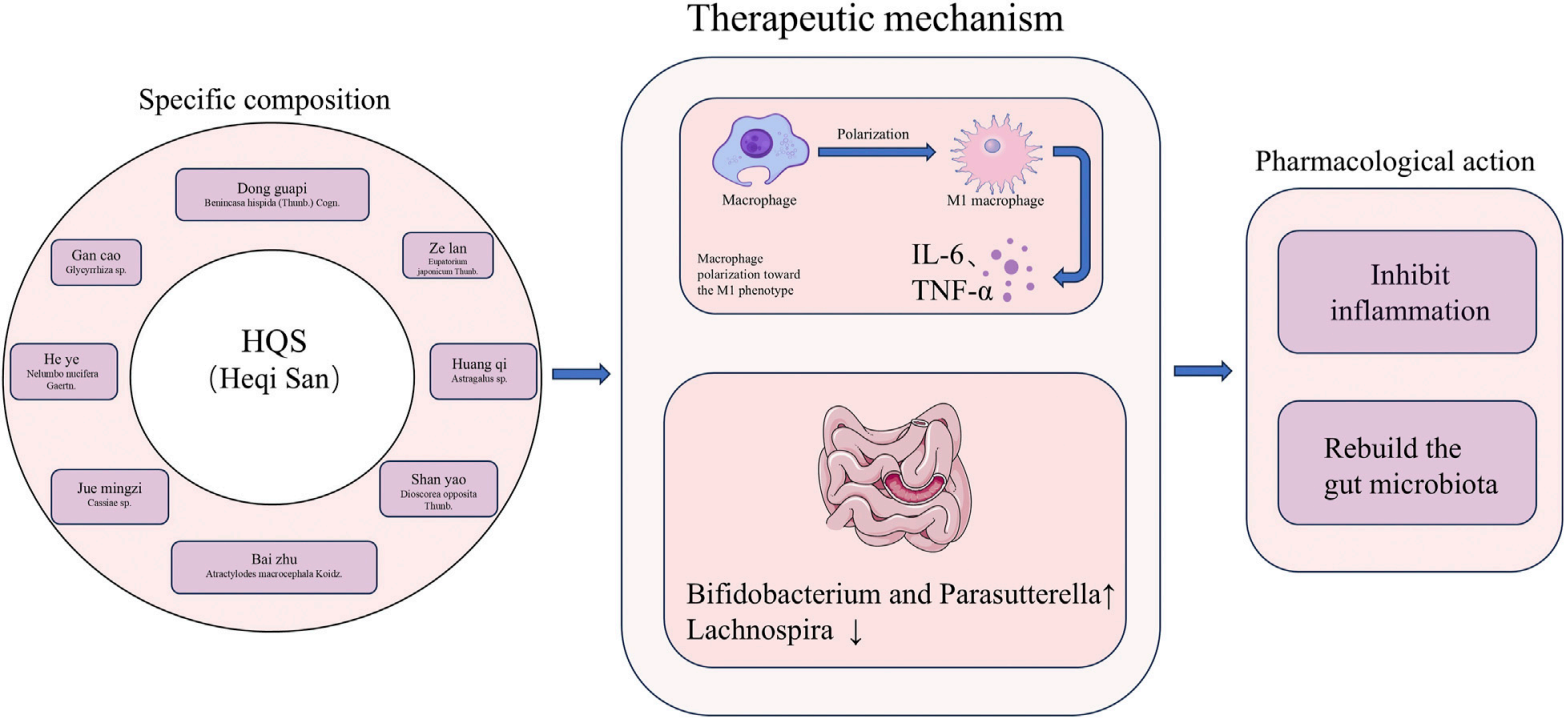
The specific composition of HeQi San and a schematic diagram of its mechanism in improving insulin resistance in polycystic ovary syndrome. (Abbreviations: IL-6: Interleukin-6, TNF-α: Tumor Necrosis Factor-α). (Astragalus sp. (The specific species was not specified in the original study. According to the Chinese Pharmacopoeia, the medicinal Astragali radix includes two species: Astragalus membranaceus (Fisch.) Bge. var.mongholicus (Bge.) Hsiao and Astragalus membranaceus (Fisch.) Bge. Cassiae sp. (The specific species was not specified in the original study. Aaccording to the Chinese Pharmacopoeia, the medicinal Cassiae semen includes Cassia obtusifolia L. and Cassia tora L. Glycyrrhiza sp. (The specific species was not specified in the original study. According to the Chinese Pharmacopoeia, the medicinal Glycyrrhizae radix et rhizoma includes Glycyrrhiza uralensis Fisch., Glycyrrhiza glabra L. and Glycyrrhiza inflata Bat)). The Chinese pinyin, Latin names, generic names, and Latin scientific names of the above traditional Chinese medicines are from the Chinese Pharmacopoeia (2020 Edition), Kew Plants of the World Online, and Kew Medicinal Plant Names Services (MPNS). The website of the Chinese Pharmacopoeia: https://ydz.chp.org.cn/#/, the website of Kew Plants of the World Online: https://powo.science.kew.org, and the website of Kew Medicinal Plant Names Services (MPNS): https://www.kew.org/science/our-science/science-services/medicinal-plant-names-services.

HEQI San (HQS) is a widely utilized TCM compound comprising He ye (Lotus leaf), Huang qi (Astragalus), Ze lan (Zeran), Jue mingzi (Cassia seed), Dong guapi (Exocarpium benincasae), Bai zhu (Atractylodes macrocephala), Shan yao (Chinese yam), and Gan cao (Licorice), all botanical drugs. A recent animal study demonstrated that HQS effectively ameliorates IR in PCOS mice through diverse mechanisms (Figure 4). HQS suppresses NF-κB-mediated M1 polarization of macrophages, thereby reducing the secretion of inflammatory cytokines like IL-6 and TNF-α, consequently mitigating the disruptive impact of inflammation on the insulin signaling pathway (Li J. et al., 2024). Furthermore, HQS enhances insulin sensitivity in PCOS mice by modulating the composition of gut microbiota (Li J. et al., 2024).

In addition to the above TCM botanical drug compound prescriptions, recent studies have also indicated that other TCM botanical drug compound prescriptions can effectively improve IR in PCOS (Supplementary Table S1). These TCM botanical drug compound prescriptions are worthy of further development and utilization (An et al., 2025; Guan et al., 2024; Huang et al., 2024a; Huang et al., 2024b; Lian et al., 2020; Liu et al., 2025; Liu et al., 2021a; Qiu et al., 2020; Su et al., 2023; Wu et al., 2022b; Xu et al., 2021; Zhang et al., 2023b; Zhang et al.,2025; Zhang and Xu, 2021; Zhao et al., 2022b; Zheng et al., 2023).

##### 5.1.1.2 TCM botanical drug metabolites

Advancements in TCM extraction and chemical structure analysis have enabled researchers to investigate the medicinal chemistry and pharmacology of TCM. Studies have revealed that certain TCM botanical drug active metabolites, including isoquinoline alkaloids and flavonoids, exhibit potential in ameliorating IR associated with PCOS.

**FIGURE 5:**
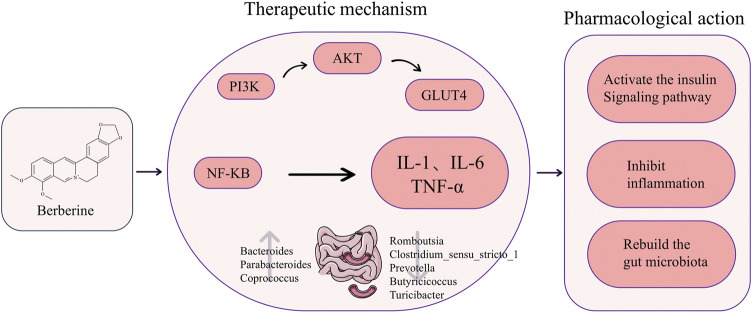
The chemical structure of berberine and a diagram of its specific mechanism in improving insulin resistance in polycystic ovary syndrome. (Abbreviations: PI3K: Phosphoinositide 3-Kinase, AKT: Protein Kinase B, GLUT4: Glucose Transporter 4, NF-KB:Nuclear Factor-kappa B, IL-1: Interleukin-1, IL-6: Interleukin-6, TNF-α: Tumor Necrosis Factor-α).

TCM plants such as Phellodendron chinense Schneid. and Coptis chinensis Franch. yield berberine, an isoquinoline quaternary ammonium alkaloid with notable biological efficacy (Wu et al., 2019; Chen et al., 2020). Its clinical utility has garnered considerable interest owing to its robust antibacterial, anti-inflammatory, and metabolic regulatory attributes, coupled with minimal side effects and a high safety profile (Kong et al., 2020; Blais et al., 2023). Recent research indicates that berberine effectively ameliorates IR in PCOS patients through diverse mechanisms, including the activation of insulin-related pathways and the suppression of aberrant inflammatory factor release (Figure 5). On the one hand, increased insulin sensitivity results from berberine’s direct impact on the insulin signaling system. Zhang N. et al. (2020) demonstrated that berberine activates the PI3K/AKT pathway, boosting insulin signaling and increasing both mRNA and protein levels of GLUT4 in ovarian tissue in a dose-dependent manner. This augmentation enhances cellular glucose transport capacity, thereby ameliorating insulin resistance. Furthermore, berberine elevates the cellular levels of phosphorylated PI3K (p-PI3K) and phosphorylated AKT (p-AKT) in the ovarian tissue of rats with PCOS, thereby activating this pathway and improving insulin sensitivity. Notably, *in vitro* experiments revealed that inhibition of either PI3K or AKT negated the beneficial effects of berberine on IR, underscoring the pivotal role of the PI3K/AKT pathway in its mechanism of action (Yu et al., 2021). On the other hand, berberine enhances insulin sensitivity by inhibiting inflammatory factors. It achieves this by suppressing the activation of MAPK and NF-κB signaling pathways, leading to reduced expression of corresponding proteins in ovarian tissues (Zhao F.-Q. et al., 2022). This action alleviates local ovarian inflammation, thereby ameliorating insulin resistance. Furthermore, berberine downregulates the expression of TLR4 and NF-κB, consequently inhibiting the secretion of inflammatory factors and ultimately improving insulin sensitivity (Shen et al., 2021a). Berberine, in addition to activating insulin-related signaling pathways and inhibiting inflammatory factors, exerts its effects on improving PCOS-related IR by modulating the gut microbiota. Shen et al., 2021b demonstrated a significant decrease in the HOMA-IR index of PCOS-IR rats treated with berberine (150 mg/kg/d by gavage for 6 weeks), accompanied by a reduction in Firmicutes abundance and an increase in Bacteroidetes levels in the gut microbiota. Similarly, Xin et al. (2024) animal studies supported the efficacy of berberine in ameliorating PCOS-IR through restructuring the gut microbiota. However, not all findings align with these positive outcomes. Wang et al. (2021) observed that neither low nor high doses of berberine significantly improved insulin resistance in PCOS-IR model mice. The divergent results among these studies suggest that the dosage and duration of berberine intervention may play pivotal roles in determining treatment efficacy.

Quercetin, a ubiquitous flavonoid metabolite in TCM botanical drugs, exhibits diverse physiological functions including anti-inflammatory and antioxidant properties. Its efficacy in treating gynecological disorders such as endometriosis underscores its importance in human health (An et al., 2022; Hipólito-Reis et al., 2022; Shah et al., 2024). Additionally, quercetin demonstrates a beneficial impact on ameliorating IR in PCOS. Li M. et al. (2023) observed a notable decrease in PM20D1 levels in a PCOS-IR rat model, concomitant with evident insulin resistance. Subsequent administration of quercitrin to these rats demonstrated an amelioration of insulin resistance through the upregulation of PM20D1 expression and activation of the PI3K/Akt insulin pathway. In a separate study, Zheng L. et al. (2022) established a DHEA-induced PCOS rat model characterized by pronounced IR and elevated levels of inflammatory markers such as IL-6 and TNF-α. Treatment with quercetin significantly alleviated inflammation and IR in the PCOS rat model (Zheng S. et al., 2022). Mechanistically, quercetin is postulated to mitigate ovarian inflammation in PCOS model rats by reducing inflammatory markers, thereby enhancing insulin sensitivity. Furthermore, quercetin has been shown to lower androgen levels in PCOS model mice while improving IR (Shah et al., 2023). A recent systematic review and meta-analysis evaluating the efficacy of quercetin in PCOS animal models revealed its significant reduction of FINS, fasting blood glucose (FBG), and HOMA-IR levels, comparable to metformin (Su et al., 2024). These findings underscore the promising therapeutic potential of quercetin in addressing PCOS-related IR.

Apart from the TCM botanical drug metabolites previously discussed, additional metabolites from TCM botanical drugs have demonstrated efficacy in ameliorating PCOS-IR (Table 1). The underlying mechanism of its action may be associated with the activation of the insulin signaling pathway and the suppression of inflammation.

**TABLE 1:** Animal studies on improving IR in PCOS with single TCM botanical drug metabolites alone.

Name	Class	Evaluation model	Mechanism of action	The literature
Cryptotanshinone	Terpenoids	HCG + Insulin induced PCOS with IR in rats	Androgen levels were reduced	Liu et al. (2024)
Curcumin	Phenolic	DHEA induced PCOS with IR in rats	Upregulated the protein level of PPAR-γ	Zhang et al. (2024c)
Curcumin	Phenolic	DHEA induced PCOS with IR model in rats	Androgen levels were reduced	Zhang et al. (2021)
Curcumin	Phenolic	DHEA induced PCOS with IR model in mice	Inhibited inflammation	Yang et al. (2024)
Curcumin	Phenolic	Letrozole induced PCOS with IR in rats	Regulated the IRS1/PI3K/GLUT4 signaling pathway	Zheng et al. (2022a)
Glycyrrhizin	Terpenoids	DHEA + HFD induced PCOS with IR in mice	Inhibited inflammation	Yang et al. (2023)
Hyperoside	Flavonoids	Letrozole + HFD induced PCOS with IR in mice	NA	Zhou et al. (2025)
Icariin	Flavonoids	Letrozole + HFD induced PCOS with IR in rats	Inhibited inflammation	Zuo et al. (2023)
Icariin	Flavonoids	Letrozole + HFD induced PCOS with IR in rats	Androgen levels were reduced	Hai et al. (2023)
Luteolin	Flavonoids	Letrozole + HFD Induced PCOS with IR in rats	Activated the PI3K/AKT signaling pathway	Huang and Zhang (2021)
Mogroside V	Terpenoids	Letrozole + HFD Induced PCOS with IR in rats	Regulated the IGF1/IGF1R pathway, Inhibited inflammation	Yang et al. (2025)
Morin	Flavonoids	Insulin + HCG induced PCOS with IR in rats	Inhibited inflammation	Yang et al. (2022c)
Naringenin	Flavonoids	DHEA induced PCOS with IR in rats	Upregulated the expression of PKGIα	Xiang et al. (2023)
Naringenin	Flavonoids	Insulin + HCG induced PCOS with IR in rats	Inhibited inflammation	Yang et al. (2022c)
Naringenin	Flavonoids	Letrozole induced PCOS with IR in rats	Remodeled the gut microbiota composition	Wu et al. (2022a)
Pachymic acid	Terpenoids	DHEA induced PCOS with IR model in mice	Inhibited inflammation, Upregulated the expression of IRS-1 and GLUT-4	Fu et al. (2020)
Pterostilbene	Stilbenoids	Letrozole induced PCOS with IR in rats	Regulated the IRS-1/PI3K/AKT signaling pathway	Foda et al. (2024)
Resveratrol	Phenolic	Letrozole + HFD induced PCOS with IR in rats	NA	Huo et al. (2023)

#### 5.1.2 Evidence from human studies

While animal experiments have offered valuable insights into the mechanisms of TCM in treating PCOS with IR, the transition from laboratory research to clinical practice encounters several obstacles. Primarily, animal models typically represent a single etiological factor, limiting their ability to fully replicate the multifaceted pathological features of human PCOS with IR, including genetic and environmental influences (Ryu et al., 2019; Frangogiannis, 2022; Kamada et al., 2022). Furthermore, animal studies tend to concentrate on short-term molecular-level effects, whereas human PCOS with IR involves intricate metabolic-reproductive axis dysregulation (Finnie and Blumbergs, 2022; Frangogiannis, 2022; Kamada et al., 2022). The prolonged disease progression and individual variability in humans may result in disparities between animal study outcomes and clinical effectiveness (Finnie and Blumbergs, 2022; Frangogiannis, 2022; Kamada et al., 2022).

Although animal models have limitations, they offer valuable insights into the effects of TCM that are pertinent to clinical research. This discussion will now shift focus to human research findings to elucidate the practical impact of TCM on improving IR in PCOS patients. By bridging basic research with clinical application, this examination aims to underscore the relevance of TCM in treating PCOS-related IR in human subjects.

##### 5.1.2.1 TCM botanical drug formulas

As previously mentioned, animal studies on PCOS with IR induced by different drugs have confirmed the significant effects of TCM botanical drug formulas on improving IR from multiple aspects. Recent human studies have further confirmed that TCM botanical drug formulas also have significant efficacy in improving IR in patients with PCOS-IR.

A recent clinical study indicated that the sole application of Dingkun Pill can effectively enhance insulin sensitivity in patients with PCOS (Deng et al., 2020). Moreover, the results of this study demonstrated that, in terms of improving insulin sensitivity and regulating lipid metabolism (reducing total cholesterol, low - density lipoprotein cholesterol, and free fatty acids), the sole use of Dingkun Pill outperforms the sole use of Diane - 35 (Deng et al., 2020). This suggests that in the treatment of PCOS, Dingkun Pill has certain application potential in improving metabolic abnormalities. Particularly for patients mainly presenting with IR and lipid metabolism disorders, it may be a treatment option worthy of attention. Another retrospective cohort study showed that the sole use of Zishen Yutai Pills can effectively improve IR in patients with PCOS, and its efficacy in improving IR in PCOS patients is similar to that of the sole use of metformin (Zhang Y. et al., 2024). Additionally, the levels of sex hormones such as LH and T in PCOS patients also improved after treatment with Zishen Yutai Pills (Zhang Y. et al., 2024). The specific drug compositions and other relevant information of the above-mentioned TCM botanical drug formulas are detailed in Table 2.

**TABLE 2:** Human studies on the improvement of IR in PCOS by TCM botanical drug formulas alone.

Name	Composition	Experimental type	Study design	Improvement of other symptoms	The extraction procedure	The literature
Dingkun Pill	Full specific composition not specified	*In vivo*	RCT	TC↓, LDL-C↓, FFA↓, Acne score↓	The specific extraction method was not specified	Deng et al. (2020)
Zishen Yutai pills	Cuscuta sp. [Convolvulaceae; Cuscutae Semen] (The specific species was not specified in the original study.According to the Chinese Pharmacopoeia, the medicinal Cuscutae Semen includes Cuscuta chinensis Lam. and Cuscuta australis R.Br.), Amomi sp. [Zingiberaceae; Amomi fructus] (the specific species was not specified in the original study; according to the Chinese Pharmacopoeia, the medicinal Amomi fructus includes Amomum villosum Lour., Amomum villosum Lour. var. xanthioides T. L. Wu et Senjen and Amomum longiligulare T. L. Wu.), Rehmannia glutinosa Libosch. [Scrophulariaceae; Rehmanniae radix], Panax ginseng C. A. Mey. [Araliaceae; Ginseng radix et rhizoma], Taxillus chinensis (DC.) Danser [Loranthaceae; Taxilli herba], *Equus asinus* Linnaeus [Equidae; Asini Corii Colla], Polygonum multiflorum Thunb. [Polygonaceae; Polygoni multiflori radix], Artemisia argyi Levl. et Van. [Asteraceae; Artemisiae argyi folium], Morinda officinalis How [Rubiaceae; Morindae officinalis radix], Atractylodes macrocephala Koidz. [Asteraceae; Atractylodis macrocephalae rhizoma], Codonopsis sp. [Campanulaceae; Codonopsis radix] (The specific species was not specified in the original study.According to the Chinese Pharmacopoeia, the medicinal Codonopsis radix includes Codonopsis pilosula (Franch.) Nannf., Codonopsis pilosula Nannf. var. modesta (Nannf.) L. T. Shen and Codonopsis tangshen Oliv.), Cervus sp. [Cervidae; Cervi cornus] (The specific species was not specified in the original study.According to the Chinese Pharmacopoeia, the medicinal Cervi cornus includes *Cervus elaphus* Linnaeus and Cervus nippon Temminck.), Lycium barbarum L. [Solanaceae; Lycii Barbari Fructus], Dipsacus asperoides C. Y. Cheng et T. M. Ai. [Dipsacaceae; RADIX DIPSACI], Eucommia ulmoides Oliv. [Eucommiaceae; Eucommiae cortex] The specific dosages (in grams) of the traditional Chinese medicines involved in this study were not clearly indicated in the original data	*In vivo*	NRCT	BMI↓, LH↓, E2↓, T↓, TCM syndrome scores↓, number of high-quality embryos↑, clinical pregnancy rate↑, embryo implantation rate↑, abortion rate↓	The specific extraction method was not specified	Zhang et al. (2024e)

##### 5.1.2.2 TCM botanical drug metabolites

In addition to animal experiments, the effectiveness of TCM botanical drug metabolites in improving PCOS with IR has also been confirmed by human studies.

Curcumin is a phenolic compound extracted from plants of Zingiberaceae, Araceae, etc. (such as Curcuma longa and Curcuma aromatica) (Garimella and Pradhan, 2024). Modern research has shown that curcumin has a wide range of pharmacological effects, including anti - inflammation, anti - oxidation, and anti - tumor effects. It can regulate multiple signaling pathways in the body and inhibit the release of inflammatory factors (Weng and Goel, 2022; Cox et al., 2022; Luo et al., 2023; Zhang J. et al., 2023). First, multiple RCTs have directly confirmed the independent efficacy of curcumin. A randomized double - blind placebo - controlled trial indicated that the sole application of curcumin in treatment can significantly improve insulin sensitivity in patients with PCOS (Jamilian et al., 2020). Another clinical study showed that the sole use of nano - curcumin can effectively improve insulin resistance in PCOS patients, but its efficacy is weaker than that of metformin (Feghhi et al., 2024). The combined application of metformin and nano - curcumin can more significantly improve IR in PCOS patients, which reflects the synergistic effect of the combined use of traditional Chinese and Western medicines (Feghhi et al., 2024). On this basis, systematic reviews and Meta - analyses based on RCTs have further strengthened the above conclusions. A systematic review and Meta - analysis based on RCTs stated that whether curcumin is used alone or on the basis of metformin, it can significantly improve IR in PCOS patients and effectively improve inflammatory markers, with no obvious adverse reactions (Shen et al., 2022). Another systematic review and Meta - analysis based on RCTs indicated that although curcumin can effectively improve IR in PCOS patients, its effectiveness in lipid regulation is still lacking (specifically, it has no significant impact on lipid parameters such as total cholesterol, LDL - C, HDL - C, and triglycerides) (Simental-Mendía et al., 2022). However, not all studies have reached consistent conclusions. Some studies have shown that although the application of curcumin can effectively reduce fasting blood glucose and dehydroepiandrosterone (DHEA) levels in PCOS patients, it has no obvious improvement on IR indicators (Heshmati et al., 2021). Combining the above research results, it can be seen that the improvement effect of curcumin on IR in PCOS patients may be related to factors such as the form of administration, the combined medication regimen, and individual differences among patients. Its specific mechanism of action and applicable conditions still need further research for clarification.

Recent studies have verified the efficacy of berberine in ameliorating IR in individuals with PCOS. A systematic review of clinical trials demonstrated that berberine administration alone effectively reduces IR in PCOS patients, albeit with a potential for mild gastrointestinal side effects (Mirzaee et al., 2021). Additionally, a network meta-analysis revealed that combining berberine with metformin yields superior improvements in IR compared to metformin monotherapy in PCOS patients (Zhao et al., 2021). These findings collectively support the significant efficacy of berberine, either as a standalone treatment or in conjunction with metformin, in enhancing IR in PCOS patients. This underscores the dual benefits of berberine in managing PCOS-related IR, positioning it as a versatile and dependable therapeutic option for addressing IR in PCOS patients.

Crocins, carotenoid compounds derived from the dried stigmas of Crocus sativus L. (saffron) in the Iridaceae family, exhibit diverse pharmacological properties, including anti-inflammatory effects and enhancement of microcirculation (Bahari et al., 2025; Hao et al., 2025). In a study by Rahimi et al. (2022), patients with PCOS were administered crocin or a placebo for 12 weeks. Results showed that compared to the placebo group, the crocin-treated group exhibited significant reductions in fasting insulin levels, HOMA-IR index, and IL-6 levels. Moreover, the crocin intervention led to a decrease in the atherosclerosis index and an increase in the cardioprotective index (Rahimi et al., 2022). These findings suggest that crocins effectively safeguard the cardiovascular system, ameliorate insulin resistance, and mitigate inflammation in women with PCOS.

The specific experimental details of the above research on the improvement of IR in PCOS patients by TCM botanical drug metabolites are summarized in Table 3.

**TABLE 3:** Human studies on the improvement of IR in PCOS by single TCM botanical drug metabolites alone.

Name	Class	Experimental type	Study design	Improvement of other symptoms	The literature
Berberine	Alkaloids	*In vivo*	Systematic review (including RCTs and NRCTs)	TT↓, FAI↓ WHR↓, SHBG↑	Mirzaee et al. (2021)
Berberine	Alkaloids	*In vivo*	Network Meta-Analysis of RCTs	TT↓, BMI↓	Zhao et al. (2021)
Crocin	Terpenoids	*In vivo*	RCT	HDL-C↑, IL-6↓	Rahimi et al. (2022)
Curcumin	Phenolic	*In vivo*	RCT	HDL↑ TC↓ LDL↓FBG↓	Jamilian et al. (2020)
Curcumin	Phenolic	*In vivo*	RCT	HDL↑, LDL↓, TC↓, TG↓	Feghhi et al. (2024)
Curcumin	Phenolic	*In vivo*	Systematic Review and Meta-analysis of RCTs	TC↓, CRP↓	Shen et al. (2022)
Curcumin	Phenolic	*In vivo*	Systematic Review and Meta-analysis of RCTs	FBG↓, FINS↓	Simental-Mendía et al. (2022)

### 5.2 Combined application of TCM botanical drug products on the basis of conventional drugs to improve IR in PCOS

#### 5.2.1 Evidence from animal studies

In animal studies investigating the enhancement of IR in PCOS through the integration of TCM with conventional pharmaceutical interventions, the limited yet emerging evidence underscores the synergistic benefits of this combined approach, delineated along two primary dimensions: TCM botanical drug formulas and TCM botanical drug metabolites. Specifically, experiments utilizing Chinese botanical drug formulas have demonstrated that the concurrent administration of the modern medication rosiglitazone with the botanical drug formulas Guizhi Fuling Pills (GFW) yields a more pronounced improvement in IR compared to rosiglitazone monotherapy (Ye et al., 2023). Furthermore, this combined regimen exhibits superior regulatory efficacy in various domains, including modulation of lipid metabolism (evidenced by reductions in total cholesterol, triglycerides, and low-density lipoprotein, alongside increases in high-density lipoprotein), amelioration of sex hormone imbalances (manifested by decreased testosterone and LH levels, and increased estradiol, progesterone, among others), and mitigation of inflammatory responses (attested by diminished levels of inflammatory markers such as CRP, IL-18, and TNF-α) (Ye et al., 2023). These findings underscore the synergistic and potentiated impact of integrating Chinese botanical drug formulas with modern pharmacotherapy in addressing the endocrine dysregulation characteristic of PCOS. Animal studies have shown promising outcomes when combining TCM botanical drug metabolites with conventional drugs. For instance, Zhang et al. (2021) investigated the impact of curcumin in conjunction with aerobic exercise on a DHEA-induced PCOS rat model. The findings revealed that this combined approach significantly ameliorated IR, leading to greater reductions in FBG, FINS, and the HOMA-IR compared to using curcumin or aerobic exercise alone (Zhang et al., 2021). Furthermore, the combined intervention exhibited superior effects in modulating sex hormone levels (such as decreasing T and LH while increasing FSH) and improving ovarian tissue pathology (including reducing atretic follicles and increasing corpora lutea count) (Zhang et al., 2021). These results suggest that integrating TCM botanical drug metabolites with standard therapy can holistically enhance various PCOS-related abnormalities through synergistic actions on multiple targets and pathways, underscoring the potential for optimizing PCOS treatment strategies.

In conclusion, despite the limited sample sizes in current animal studies, they consistently indicate that combining TCM botanical drug formulas or metabolites with conventional drugs can synergistically enhance the amelioration of IR in PCOS. This offers an experimental foundation for future clinical translation.

#### 5.2.2 Evidence from human studies

##### 5.2.2.1 TCM botanical drug formulas

In recent years, researchers have investigated the potential benefits of augmenting the effectiveness of conventional modern medicine treatments for IR in PCOS by incorporating TCM botanical drug formulas.

A prospective RCT demonstrated that combining Diane-35 with Dingkun Pill, in addition to Diane-35 alone, offers greater benefits in enhancing IR (Deng et al., 2020). Furthermore, the study revealed that combining Dingkun Pill with Diane-35, beyond Dingkun Pill alone, is more effective in decreasing total T levels and DHEAS, while increasing the rate of menstrual recovery (Deng et al., 2020). These findings underscore the synergistic and complementary therapeutic effects of the combined approach, providing a more comprehensive intervention targeting the multifaceted pathological aspects of PCOS. Shi et al. (2022) demonstrated that combining Jiawei Huanglian Wendan Decoction (JHWD) with metformin and Diane-35 in the treatment of PCOS patients with IR significantly enhances the patients’ IR status. This combined therapy also exhibits benefits in ameliorating additional parameters such as blood lipid levels (TC, triglycerides, LDL) and inflammatory markers (TNF-α, IL-6, IL-1) (Shi et al., 2022). These findings suggest that integrating TCM botanical drug formulas with standard treatment can comprehensively address metabolic irregularities and clinical manifestations in PCOS patients with IR.

Further clinical evidence reinforces the synergistic impact of integrating TCM botanical drug formulas with conventional modern medicine for managing IR in PCOS patients. Various studies, employing diverse experimental designs and outcome measures, consistently demonstrate the benefits of this combined approach in enhancing insulin sensitivity, managing metabolic irregularities, and addressing associated clinical manifestations. Specific details regarding drug compositions and trial characteristics are outlined in Table 4.

**TABLE 4:** Human studies on the combined application of TCM botanical drug formulas in improving IR in PCOS.

Name	Composition	Experimental type	Study design	Other symptoms with more significant improvement in TCM combination versus conventional therapy alone	Treatment interventions	The extraction procedure	The literature
Cangfu Daotan Decoction	Atractylodes sp. [10 g; Asteraceae; Atractylodis rhizoma] (The specific species was not specified in the original study.According to the Chinese Pharmacopoeia, the medicinal Atractylodis rhizoma includes Atractylodes lancea (Thunb.) DC. and Atractylodes chinensis (DC.) Koidz.), Arisaema erubescens (Wall.) Schott, Arisaema heterophyllum Blume, Arisaema amurense Maxim. [10 g; Araceae; Rhizoma Arisaematis], Acorus tatarinowii Schott [10 g; Araceae; Acori tatarinowii rhizoma], Pinellia ternata (Thunb.) Makino [10 g; Araceae; Pinelliae rhizoma], Gleditsia sinensis Lam. [10 g; Fabaceae; Gleditsiae spina], Angelica sinensis (Oliv.) Diels [10 g; Apiaceae; Angelicae sinensis radix], Cyperus rotundus L. [15g; Cyperaceae; Cyperi rhizoma], Poria cocos (Schw.) Wolf [15 g; Polyporaceae; Poria], Salvia miltiorrhiza Bunge [15 g; Lamiaceae; Salviae miltiorrhizae radix et rhizoma], Rehmannia glutinosa Libosch. [15 g; Scrophulariaceae; Rehmanniae radix], Cuscuta sp. [20 g; Convolvulaceae; Cuscutae Semen] (The specific species was not specified in the original study. According to the Chinese Pharmacopoeia, the medicinal Cuscutae Semen includes Cuscuta chinensis Lam. and Cuscuta australis R.Br.), Citrus reticulata Blanco [6 g; Rutaceae; Citri reticulatae pericarpium]	*In vivo*	NRCT	E2↑ LH↓ FSH↓ T↓ TCHO↓ TG↓ LDL-C↓ HDL-C↑ Pregnancy rate↑	Drospirenone and ethinylestradiol tablets (II)/Drospirenone and ethinylestradiol tablets (II) + CDD	These Chinese herbal medicines were decocted with 400 mL of clear water	Fu et al. (2024b)
Dingkun Pill	Full specific composition not specified	*In vivo*	RCT	QUICKI↑	Dingkun Pill/Diane-35/Dingkun Pill + Diane-35	The specific extraction method was not specified	Deng et al. (2020)
Guizhi Fuling formula	Prunus sp. [Rosaceae; Persicae Semen] (The specific species was not specified in the original study.According to the Chinese Pharmacopoeia, the medicinal Persicae Semen includes Prunus persica (L.) Batsch and Prunus davidiana (Carr.) Franch.), Cinnamomum cassia (L.) J. Presl [Lauraceae; Cinnamomi cassiae ramulus], Poria cocos (Schw.) Wolf [Polyporaceae; Poria], Paeonia lactiflora Pall. [Ranunculaceae; Paeoniae Alba Radix], Paeonia suffruticosa Andr. [Ranunculaceae; Paeoniae suffruticosae cortex] The specific dosages (in grams) of the traditional Chinese medicines involved in this study were not clearly indicated in the original data	*In vivo*	Meta-analysis of RCTs	Ovulation rate ↑ pregnancy rate ↑ FSH ↓ T ↓ LH ↓	conventional medicine (Clomiphene citrate, ECA, Metformin, Pioglitazone)/conventional medicine + GZFL	The specific extraction method was not specified	Rong et al. (2022)
Jiawei Huanglian-Wendan decoction	Coptis sp. [6 g; Ranunculaceae; Coptidis Rhizoma] (The specific species was not specified in the original study. According to the Chinese Pharmacopoeia, the medicinal Coptidis Rhizoma includes Coptis chinensis Franch., Coptis deltoidea C. Y. Cheng et Hsiao and Coptis teeta Wall.), Scutellaria baicalensis Georgi [10 g; Lamiaceae; Scutellariae radix], Bambusa sp. [15 g; Poaceae; Caulis Bambusae in Taeniam] (The specific species was not specified in the original study. According to the Chinese Pharmacopoeia, the medicinal Caulis Bambusae in Taeniam includes Bambusa tuldoides Munro, Sinocalamus beecheyanus (Munro) McClure var. pubescens P.F.Li and Phyllostachys nigra (Lodd.) Munro var. henonis (Mitf.) Stapf ex Rendle.), Citrus sp. [12 g; Rutaceae; Aurantii Fructus Immaturus] (The specific species was not specified in the original study. According to the Chinese Pharmacopoeia, the medicinal Aurantii Fructus Immaturus includes Citrus aurantium L. and Citrus sinensis Osbeck.), Poria cocos (Schw.) Wolf [15 g; Polyporaceae; Poria], Citrus reticulata Blanco [12 g; Rutaceae; Citri reticulatae pericarpium], Cyperus rotundus L. [12 g; Cyperaceae; Cyperi rotundi radix et rhizoma], Pinellia ternata (Thunb.) Makino [10 g; Araceae; Pinelliae rhizoma], Salvia miltiorrhiza Bunge [12 g; Lamiaceae; Salviae miltiorrhizae radix et rhizoma], Angelica sinensis (Oliv.) Diels [12 g; Apiaceae; Angelicae sinensis radix], Cuscuta sp. [24 g; Convolvulaceae; Cuscutae Semen] (The specific species was not specified in the original study.According to the Chinese Pharmacopoeia, the medicinal Cuscutae Semen includes Cuscuta chinensis Lam. and Cuscuta australis R.Br.), Atractylodes macrocephala Koidz. [15 g; Asteraceae; Atractylodis macrocephalae rhizoma]	*In vivo*	RCT	Normal menstrual cycle recovery rate ↑ ovulation ↑ FBG ↓ TC ↓ TG ↓ LDL-C ↓ TNF-α ↓ IL-6 ↓ IL-1 ↓	Metformin + Diane-35/Metformin + Diane-35 + JHWD	Herbs were boiled in water for 30 min and condensed into 300 mL decoction	Shi et al. (2022)
Xiao Yao San	Full specific composition not specified	*In vivo*	Meta-analysis of RCTs	Ovulation rate ↑ Pregnancy rate ↑	conventional medicine (Metformin, Letrozole, Menotrophin, Tamoxifen, HCG, HMG, Ethinylestradiol, Cyproterone)/conventional medicine + XYS	The specific extraction method was not specified	Zhou et al. (2023)
Zishen Yutai pills	Cuscuta sp. [Convolvulaceae; Cuscutae Semen] (The specific species was not specified in the original study.According to the Chinese Pharmacopoeia, the medicinal Cuscutae Semen includes Cuscuta chinensis Lam. and Cuscuta australis R.Br.), Amomi sp. [Zingiberaceae; Amomi fructus] (the specific species was not specified in the original study; according to the Chinese Pharmacopoeia, the medicinal Amomi fructus includes Amomum villosum Lour., Amomum villosum Lour. var. xanthioides T. L. Wu et Senjen and Amomum longiligulare T. L. Wu.), Rehmannia glutinosa Libosch. [Scrophulariaceae; Rehmanniae radix], Panax ginseng C. A. Mey. [Araliaceae; Ginseng radix et rhizoma], Taxillus chinensis (DC.) Danser [Loranthaceae; Taxilli herba], *Equus asinus* Linnaeus [Equidae; Asini Corii Colla], Polygonum multiflorum Thunb. [Polygonaceae; Polygoni multiflori radix], Artemisia argyi Levl. et Van. [Asteraceae; Artemisiae argyi folium], Morinda officinalis How [Rubiaceae; Morindae officinalis radix], Atractylodes macrocephala Koidz. [Asteraceae; Atractylodis macrocephalae rhizoma], Codonopsis sp. [Campanulaceae; Codonopsis radix] (The specific species was not specified in the original study.According to the Chinese Pharmacopoeia, the medicinal Codonopsis radix includes Codonopsis pilosula (Franch.) Nannf., Codonopsis pilosula Nannf. var. modesta (Nannf.) L. T. Shen and Codonopsis tangshen Oliv.), Cervus sp. [Cervidae; Cervi cornus] (The specific species was not specified in the original study.According to the Chinese Pharmacopoeia, the medicinal Cervi cornus includes *Cervus elaphus* Linnaeus and Cervus nippon Temminck.), Lycium barbarum L. [Solanaceae; Lycii Barbari Fructus], Dipsacus asperoides C. Y. Cheng et T. M. Ai. [Dipsacaceae; RADIX DIPSACI], Eucommia ulmoides Oliv. [Eucommiaceae; Eucommiae cortex] The specific dosages (in grams) of the traditional Chinese medicines involved in this study were not clearly indicated in the original data	*In vivo*	NRCT	BMI↓, FPG↓, FIN↓, HOMA-IR↓, LH↓, T↓, number of obtained eggs↑, number of high-quality embryos↑, clinical pregnancy rate↑, embryo implantation rate↑, abortion rate↓	metformin/ZSYTP/metformin + ZSYTP	The specific extraction method was not specified	Zhang et al. (2024e)

##### 5.2.2.2 TCM botanical drug metabolites

Puerarin, an isoflavone derived from the roots of Pueraria lobata, a leguminous plant in traditional Chinese medicine, exhibits various pharmacological properties such as anti-inflammatory, IR improvement, and immunomodulatory effects (Wang S. et al., 2020; Wei et al., 2024; Zhang D. et al., 2024; Wu Q. et al., 2025). It is clinically utilized in treating cardiovascular and cerebrovascular diseases, diabetes, and its complications, as well as coronary heart disease (Wang et al., 2020b). A clinical trial involving Chinese women with PCOS demonstrated that supplementing the standard treatment of Diane-35 and metformin with puerarin effectively enhances insulin sensitivity in non-obese PCOS patients (Li et al., 2021). Additionally, the study revealed that puerarin supplementation elevates SHBG levels while reducing testosterone concentrations, indicating its potential in ameliorating HA in PCOS patients and supporting its adjunctive role in PCOS management (Li et al., 2021).

Multiple studies have confirmed the supplementary effectiveness of various TCM botanical drug metabolites, beyond puerarin, in ameliorating IR in individuals with PCOS. These TCM botanical drug metabolites engage in regulating metabolic disturbances associated with PCOS through diverse mechanisms, notably enhancing insulin sensitivity and exhibiting synergistic actions in modulating hormone profiles. Detailed experimental findings are outlined in Table 5.

**TABLE 5:** Human studies on the combined application of TCM botanical drug metabolites to improve IR in PCOS.

Name	Class	Experimental type	Study design	Other symptoms with more significant improvement in TCM combination versus conventional therapy alone	Treatment interventions	The literature
Berberine	Alkaloids	*In vivo*	Systematic review (including RCTs and NRCTs)	TT↓ FAI↓ WHR↓ SHBG↑	conventional medicine (Metformin, CPA, Letrozole)/conventional medicine + Berberine	Mirzaee et al. (2021)
Curcumin	Phenolic	*In vivo*	Systematic Review and Meta-analysis of RCTs	BMI↓ CRP↓	Curcumin + Metformin/Metformin	Shen et al. (2022)
Curcumin	Phenolic	*In vivo*	Systematic Review and Meta-analysis of RCTs	FBG↓ FINS↓	Curcumin + Metformin/Metformin	Simental-Mendía et al. (2022)
Puerarin	Flavonoids	*In vivo*	RCT	SHGB↑ T↓ TC↓	Diane-35 + Metformin/Diane-35 + Metformin + Puerarin	Li et al. (2021)

## 6 Conclusion and prospects

This review validates the substantial therapeutic efficacy of TCM in ameliorating IR among with PCOS through both experimental and clinical investigations. Furthermore, it demonstrates that TCM can enhance and manage IR in PCOS patients through diverse mechanisms. Nevertheless, extant studies exhibit certain limitations. Primarily, the majority of current clinical investigations suffer from restricted geographical coverage in patient recruitment and relatively modest sample sizes, thereby necessitating further validation of the generalizability of the findings. Additionally, the follow-up durations in most clinical trials are brief or nonexistent, precluding the assessment of the enduring effects and safety profile of TCM in mitigating insulin resistance. However, animal models for PCOS often rely on hormone induction and high-fat diets, deviating from the natural disease progression in humans. This discrepancy hinders the accurate replication of the complex clinical pathology. In the realm of TCM research, challenges arise not only from the variability in the quality and composition of medicinal materials across different batches but also from the absence of standardized dosing regimens. Discrepancies in the dosage, frequency of administration, and duration of treatment with TCM monomers or compounds are commonly observed in animal studies, hindering the comparability of research findings and compromising the reliability and reproducibility of conclusions. This lack of standardization, spanning from the sourcing of medicinal materials to dosing protocols, not only complicates inter-study comparisons but also undermines the interpretability and clinical applicability of research outcomes. Additionally, the predominant use of rats and mice in research overlooks the potential benefits of employing animal models more closely resembling human physiology, such as non-human primates. Future investigations should prioritize large-scale, multi-center randomized clinical trials with extended follow-up periods. Standardizing the dose-response relationship of TCM is imperative. Emphasizing the selection of animal models that mirror human physiological characteristics can enhance the fidelity of PCOS research and facilitate a more nuanced exploration of dose-response dynamics. These efforts will enrich the therapeutic armamentarium for PCOS-relate IR.

## Glossary

AhR: aryl hydrocarbon receptor
AKT: Protein Kinase B
AR: androgen receptor
BM: I, body mass index
CCR5: C-C chemokine receptor 5
CFD: Cangfu Daotan Decoction
circRNAs: circular RNAs
DHEA: Dehydroepiandrosterone
DHEAS: Dehydroepiandrosterone sulfate
ERK: Extracellular Signal-Regulated Kinase
FBG: fasting blood glucose
FINS: fasting insulin
FSH: Follicle-Stimulating Hormone
FXR: farnesoid X receptor
GFW: Guizhi Fuling Pills
GLP-1: Glucagon-Like Peptide-1
GLUT4: glucose transporter4
GnRH: gonadotropin-releasing hormone
GPX4: Glutathione Peroxidase 4
GV: germinal vesicle
HA: Hyperandrogenism
HDL-C: High-Density Lipoprotein Cholesterol
HI: hyperinsulinemia
HOMA-IR: Homeostatic Model Assessment of Insulin Resistance
HQS: HEQI San
IκBα: Inhibitor of Nuclear Factor-kappa B alpha
IKK: IκB Kinase
IKKβ: IκB Kinase beta
IL-1: Interleukin-1
IL-1β: Interleukin-1 beta
IL-6: interleukin-6
IL-22: interleukin-22
lncRNAs: long-stranded non-coding RNAs
IR: insulin resistance
IRS: Insulin Receptor Substrate
LCN-2: Lipocalin-2
LDL-C: Low-Density Lipoprotein Cholesterol
LH: luteinizing hormone
LPS: lipopolysaccharide
MAPK: mitogen-activated protein kinase
miRNA: microRNA
mRNA: Messenger RNA
mtDNA: mitochondrial DNA
mTORC2: mammalian target of rapamycin complex 2
NETs: neutrophil extracellular traps
NF-κ: B, Nuclear Factor-kappa B
NK: natural killer
Nrf2: Nuclear Factor Erythroid 2-Related Factor 2
OM: oligomenorrhea (OM)
p-AKT: phosphorylated AKT
PCOS: Polycystic ovary syndrome
PCR: polymerase chain reaction
PDK1: Phosphatidylinositol-dependent kinase-1
pgWAT: perigonadal white adipose tissue
PIP2: phosphatidylinositol 4,5 bisphosphate
PIP3: phosphatidylinositol 3,4,5 trisphosphate
PI3K: Phosphatidylinositol 3-Kinase
p-PI3K: phosphorylated PI3K
Ras: Rat Sarcoma Viral Oncogene Homolog
RCT: Randomized Controlled Trial
ROS: reactive oxygen species
SA: secondary amenorrhea
SHGB: sex hormone-binding globulin
SNP: Single Nucleotide Polymorphism
T: Testosterone
TC: Total Cholesterol
TCM: Traditional Chinese medicine
TGF-β1: transforming growth factor-β1
TNF-α: tumor necrosis factor-α
TNFR: Tumor Necrosis Factor Receptor
TRADD: Tumor Necrosis Factor Receptor-Associated Death Domain Protein
TRAF2: Tumor Necrosis Factor Receptor-Associated Factor 2
