# Supplementary material for: Research progress on the pathogenesis, clinical impact, and traditional Chinese medicine treatment of polycystic ovary syndrome complicated by insulin resistance
Source: Front Pharmacol. 2025 Sep 11;16:1661806. doi: 10.3389/fphar.2025.1661806 (PMC12461154; doi:10.3389/fphar.2025.1661806)
Supplement: Supplementary file 1 [file Table1.docx]

| Name | Composition | Evaluation model | The effect mechanism | The extraction procedure | The literature |
| --- | --- | --- | --- | --- | --- |
| Baogui  Capsule | Epimedium sp. [Berberidaceae; Epimedii folium] (The specific species was not specified in the original study.According to the Chinese Pharmacopoeia, the medicinal Epimedii folium includes Epimedium brevicornu Maxim., Epimedium sagittatum (Sieb. et Zucc.) Maxim., Epimedium pubescens Maxim. and Epimedium koreanum Nakai.), Polygonatum sp. [Asparagaceae; Polygonati rhizoma] (the specific species was not specified in the original study; according to the Chinese Pharmacopoeia, the medicinal Polygonati rhizoma includes Polygonatum sibiricum Redouté, Polygonatum kingianum Coll. et Hemsl. and Polygonatum cyrtonema Hua.), Psoralea corylifolia L. [Fabaceae; Psoraleae fructus], Testudo sinensis Gray [Testudinidae; Testudinis carapax et plastrum], Rehmannia glutinosa Libosch. [Scrophulariaceae; Rehmanniae radix], Anemarrhena asphodeloides Bunge [Asparagaceae; Anemarrhenae rhizoma], Angelica sinensis (Oliv.) Diels [Apiaceae; Angelicae sinensis radix], Prunus sp. [Rosaceae; Persicae Semen] (The specific species was not specified in the original study.According to the Chinese Pharmacopoeia, the medicinal Persicae Semen includes Prunus persica (L.) Batsch and Prunus davidiana (Carr.) Franch.), Acorus tatarinowii Schott [Araceae; Acori tatarinowii rhizoma], Polygonum cuspidatum Sieb. et Zucc. [Polygonaceae; Polygoni cuspidati rhizoma et radix], Verbena officinalis L. [Verbenaceae; Verbenae herba], Ophiopogon japonicus (L. f.) Ker-Gawl. [Asparagaceae; Ophiopogonis radix]  The specific dosages (in grams) of the traditional Chinese medicines involved in this study were not clearly indicated in the original data. | LETROZOLE induced PCOS with IR in rats | Upregulated GLUT4, downregulated the gene expression of the IKKβ/NF-κB/SOCS3 pathway to block inflammation | The specific extraction method was not specified. | (Lian et al., 2020) |
| Bailing  Capsule | Ophiocordyceps sinensis (Berk.) G.H. Sung et al. [Ophiocordycipitaceae; Cordyceps sinensis]  The specific dosages (in grams) of the traditional Chinese medicines involved in this study were not clearly indicated in the original data. | DHEA and high - fat and sugar diet - induced PCOS with IR in mice | Inhibited the lipopolysaccharide-Toll-like receptor 4 inflammatory pathway | The specific extraction method was not specified. | (Guan et al., 2024) |
| Bushen  Huoluo  Decoction | Rehmannia glutinosa Libosch. [Scrophulariaceae; Rehmanniae radix], Angelica sinensis (Oliv.) Diels [Apiaceae; Angelicae sinensis radix] Ligusticum chuanxiong Hort. [Apiaceae; Chuanxiong Rhizoma], Paeonia lactiflora Pall. [Ranunculaceae; Paeoniae Alba Radix], Ligustrum lucidum Ait. [Oleaceae; Ligustri Lucidi Fructus], Eclipta prostrata L. [Asteraceae; Ecliptae Herba], Cuscuta sp. [Convolvulaceae; Cuscutae Semen] (The specific species was not specified in the original study.According to the Chinese Pharmacopoeia, the medicinal Cuscutae Semen includes Cuscuta chinensis Lam. and Cuscuta australis R.Br.), Leonurus japonicus Houtt. [Lamiaceae; Leonuri Fructus], Rubus chingii Hu [Rosaceae; Rubi Fructus], Epimedium sp. [Berberidaceae; Epimedii folium] (The specific species was not specified in the original study.According to the Chinese Pharmacopoeia, the medicinal Epimedii folium includes Epimedium brevicornu Maxim., Epimedium sagittatum (Sieb. et Zucc.) Maxim., Epimedium pubescens Maxim. and Epimedium koreanum Nakai.), Prunus sp. [Rosaceae; Persicae Semen] (The specific species was not specified in the original study.According to the Chinese Pharmacopoeia, the medicinal Persicae Semen includes Prunus persica (L.) Batsch and Prunus davidiana (Carr.) Franch.), Carthamus tinctorius L. [Asteraceae; Carthami Flos], Liquidambar formosana Hance [Altingiaceae; Liquidambaris Fructus], Achyranthes bidentata Blume [Amaranthaceae; Achyranthis Bidentatae Radix]  The specific dosages (in grams) of the traditional Chinese medicines involved in this study were not clearly indicated in the original data. | DHEA-induced PCOS with IR in rats | Inhabited inflammation | The specific extraction method was not specified. | (Huang et al., 2024b) |
| Bushen  Tianjing  Formula | Rehmannia glutinosa Libosch. [20g; Scrophulariaceae; Rehmanniae radix], Ligustrum lucidum Ait. [20g; Oleaceae; Ligustri Lucidi Fructus], Rubus chingii Hu [10g; Rosaceae; Rubi Fructus], Cuscuta australis R. Br. [15g; Convolvulaceae; Cuscuta australis semen], Psoralea corylifolia L. [15g; Fabaceae; Psoraleae fructus], Astragalus membranaceus (Fisch.) Bge. var. mongholicus (Bge.) Hsiao [10g; Fabaceae; Astragali radix], Salvia miltiorrhiza Bunge [10g; Lamiaceae;Salviae miltiorrhizae radix et rhizoma] | testosterone propionate induced PCOS with IR in rats | Activated the PI3K/AKT insulin signaling pathway | The herbs of BSTJF were boiled with pure water at 100 °C for 1.5 hours to obtain the aqueous extract. | (Zhang et al., 2023) |
| Cangfu  daotan  Decoction | Atractylodes sp. [15g; Asteraceae; Atractylodis rhizoma] (The specific species was not specified in the original study.According to the Chinese Pharmacopoeia, the medicinal Atractylodis rhizoma includes Atractylodes lancea (Thunb.) DC. and Atractylodes chinensis (DC.) Koidz.), Cyperus rotundus L. [10g; Cyperaceae;Cyperi rhizoma], Pinellia ternata (Thunb.) Makino [9g; Araceae;Pinelliae rhizoma], Citrus reticulata Blanco [6g; Rutaceae;Citri reticulatae pericarpium], Poria cocos (Schw.) Wolf [12g; Polyporaceae; Poria], Arisaema sp. [6g; Araceae; Arisaematis rhizoma] (The specific species was not specified in the original study. According to the Chinese Pharmacopoeia, the medicinal Arisaematis Rhizoma includes three species: Arisaema heterophyllum Blume, Arisaema erubescens (Wall.) Schott, and Arisaema amurense Maxim.), Astragalus sp. [15g; Fabaceae; Astragali radix] (The specific species was not specified in the original study.According to the Chinese Pharmacopoeia, the medicinal Astragali radix includes two species: Astragalus membranaceus (Fisch.) Bge.var.mongholicus(Bge.) Hsiao and Astragalus membranaceus (Fisch.) Bge., Citrus × aurantium L. [10g; Rutaceae;Aurantii fructus immaturus], Codonopsis sp. [15g; Campanulaceae; Codonopsis radix] (The specific species was not specified in the original study.According to the Chinese Pharmacopoeia, the medicinal Codonopsis radix includes Codonopsis pilosula (Franch.) Nannf., Codonopsis pilosula Nannf. var. modesta (Nannf.) L. T. Shen and Codonopsis tangshen Oliv.), Epimedium sp. [15g; Berberidaceae; Epimedii folium] (The specific species was not specified in the original study.According to the Chinese Pharmacopoeia, the medicinal Epimedii folium includes Epimedium brevicornu Maxim., Epimedium sagittatum (Sieb. et Zucc.) Maxim., Epimedium pubescens Maxim. and Epimedium koreanum Nakai.), Crataegus sp. [30g;Rosaceae; Crataegi fructus] (The specific species was not specified in the original study.According to the Chinese Pharmacopoeia, the medicinal Crataegi fructus includes Crataegus pinnatifida Bunge and Crataegus pinnatifida Bunge var. major N. E. Br.), Salvia miltiorrhiza Bunge[20g;Lamiaceae;Salviae miltiorrhizae radix et rhizoma],Glycyrrhiza sp. [5g;Fabaceae; Glycyrrhizae radix et rhizoma] (The specific species was not specified in the original study.According to the Chinese Pharmacopoeia, the medicinal Glycyrrhizae radix et rhizoma includes Glycyrrhiza uralensis Fisch., Glycyrrhiza glabra L. and Glycyrrhiza inflata Bat.), Sinapis alba L.[10g;Brassicaceae; Sinapis albae semen],Gallus gallus domesticus Brisson [30g;Phasianidae; Endothelii corneum gigeriae galli], Gleditsia sinensis Lam.[10g;Fabaceae; Gleditsiae spina] | Letrozole + HFD induced PCOS with IR in rats | Activated the PI3K/AKT insulin signaling pathway | The specific extraction method was not specified. | (Wang et al., 2020) |
| Cangfu  daotan  Decoction | Cyperus rotundus L. [10g; Cyperaceae; Cyperi rotundi radix et rhizoma], Citrus reticulata Blanco [6g; Rutaceae;Citri reticulatae pericarpium], Atractylodes lancea (Thunb.) DC. [15g; Asteraceae;Atractylodis rhizoma], Pinellia ternata (Thunb.) Makino [9g; Araceae;Pinelliae rhizoma], Poria cocos (Schw.) Wolf [12g; Polyporaceae; Poria], Arisaema erubescens (Wall.) Schott [6g; Araceae; Arisaematis erubentis tuber], Astragalus membranaceus (Fisch.) Bunge. [15g; Fabaceae; Astragali membranacei radix], Gleditsia sinensis Lam. [10g; Fabaceae; Gleditsiae spina] | Letrozole + HFD induced PCOS with IR in rats | Inhibited inflammation | Dissolved in heated (60°C) deionized water to obtain a 3.0 g/mL stock solution and stored at 4°C. | (Jiang et al., 2022) |
| Fufang  Zhenzhutiaozhi  Formula | Ligustrum lucidum Ait. [Oleaceae; Ligustri Lucidi Fructus], Atractylodes macrocephala Koidz. [Asteraceae; Atractylodis macrocephalae rhizoma], Coptis chinensis Franch. [Ranunculaceae; Coptidis rhizoma], Citrus medica L. [Rutaceae; Citri medicae fructus, Panax notoginseng (Burkill) F.H.Chen [Araliaceae; Notoginseng radix et rhizoma], Salvia miltiorrhiza Bunge [Lamiaceae; Salviae miltiorrhizae radix et rhizoma], Eucommia ulmoides Oliv. [Eucommiaceae; Eucommiae cortex], Cirsium japonicum (Thunb.) Fisch. ex-DC [Asteraceae; Cirsii japonici herba et radix]  The specific dosages (in grams) of the traditional Chinese medicines involved in this study were not clearly indicated in the original data. | Letrozole + HFD induced PCOS with IR in mice | Androgen levels were reduced | A combination of alcohol extraction and water extraction was employed to extract components from 8 kinds of herbs. The weighed herbal powder was extracted with methanol in an ultrasonic bath (300 W, 40 kHz, 40 min). The extract was filtered through a 0.45 μm Nylon 66 membrane, and the concentrations of 15 bioactive compounds were determined by HPLC for quality control. Voucher specimens were deposited (No. GDPUZYY, 20080901-8). | (Xu et al., 2021) |
| Guizhi  Fuling  Wan | Cinnamomum cassia (L.) J. Presl [Lauraceae; Cinnamomi cassiae ramulus], Poria cocos (Schw.) Wolf [Polyporaceae; Poria], Paeonia suffruticosa Andr. [Ranunculaceae; Paeoniae suffruticosae cortex], Prunus persica (L.) Batsch [Rosaceae; Persicae semen], Paeonia veitchii Lynch [Ranunculaceae; Paeoniae veitchii radix]  The specific dosages (in grams) of the traditional Chinese medicines involved in this study were not clearly indicated in the original data. | Letrozole + HFD induced PCOS with IR in rats. | Activated the PI3K/AKT/mTOR signaling pathway | The specific extraction method was not specified. | (Liu et al., 2021) |
| Guizhi  Fuling  Wan | Cinnamomum cassia (L.) J. Presl [Lauraceae; Cinnamomi cassiae ramulus], Poria cocos (Schw.) Wolf [Polyporaceae; Poria], Paeonia lactiflora Pall. [Ranunculaceae; Paeoniae Alba Radix], Paeonia suffruticosa Andr. [Ranunculaceae; Paeoniae suffruticosae cortex], Prunus sp. [Rosaceae; Persicae Semen] (The specific species was not specified in the original study.According to the Chinese Pharmacopoeia, the medicinal Persicae Semen includes Prunus persica (L.) Batsch and Prunus davidiana (Carr.) Franch.)  The specific dosages (in grams) of the traditional Chinese medicines involved in this study were not clearly indicated in the original data. | DHEA-induced PCOS with IR in rats | Activated the PI3K/AKT insulin signaling pathway、inhibited inflammation | The specific extraction method was not specified. | (Ye et al., 2023) |
| Heqi San | Nelumbo nucifera Gaertn. [Nelumbonaceae; Nelumbinis Folium], Astragalus sp. [Fabaceae; Astragali radix] (The specific species was not specified in the original study.According to the Chinese Pharmacopoeia, the medicinal Astragali radix includes two species: Astragalus membranaceus (Fisch.) Bge.var.mongholicus(Bge.) Hsiao and Astragalus membranaceus (Fisch.) Bge., Eupatorium japonicum Thunb. [Asteraceae; Eupatorii japonicum herba], Cassiae sp. [Fabaceae; Cassiae semen] (The specific species was not specified in the original study.Aaccording to the Chinese Pharmacopoeia, the medicinal Cassiae semen includes Cassia obtusifolia L. and Cassia tora L., Benincasa hispida (Thunb.) Cogn. [Cucurbitaceae; Benincasae exocarpium], Atractylodes macrocephala Koidz. [Asteraceae; Atractylodis macrocephalae rhizoma], Dioscorea opposita Thunb. [Dioscoreaceae; Dioscoreae oppositae rhizoma], Glycyrrhiza sp. [Fabaceae; Glycyrrhizae radix et rhizoma] (The specific species was not specified in the original study.According to the Chinese Pharmacopoeia, the medicinal Glycyrrhizae radix et rhizoma includes Glycyrrhiza uralensis Fisch., Glycyrrhiza glabra L. and Glycyrrhiza inflata Bat.)  The specific dosages (in grams) of the traditional Chinese medicines involved in this study were not clearly indicated in the original data. | DHEA + HFD induced PCOS with IR in mice | inhibited inflammation、Remodeled the gut microbiota composition | The specific extraction method was not specified. | (Li et al., 2024a) |
| Hehuanyin  Decoction | Albizia julibrissin Durazz. [Fabaceae; Albiziae cortex], Ampelopsis japonica (Thunb.) Makino [Vitaceae; Ampelopsis radix]  The specific dosages (in grams) of the traditional Chinese medicines involved in this study were not clearly indicated in the original data. | Letrozole + HFD induced PCOS with IR in rats | Activated the PI3K/AKT insulin signaling pathway | The mixture was heated in an automatic medicine pot at 100 °C for 2 h and then filtered through a gauze. Subsequently, the extract was concentrated twice using a rotary evaporator to a concentration of 1.75 g/ml and stored at -20 °C for subsequent experiments. | (Wu et al., 2022) |
| Jianpi  Yishenhuazhuo  Formulation | Astragalus sp. [30g; Fabaceae; Astragali radix] (The specific species was not specified in the original study.According to the Chinese Pharmacopoeia, the medicinal Astragali radix includes two species: Astragalus membranaceus (Fisch.) Bge.var.mongholicus(Bge.) Hsiao and Astragalus membranaceus (Fisch.) Bge., Codonopsis sp. [15g; Campanulaceae; Codonopsis radix] (The specific species was not specified in the original study.According to the Chinese Pharmacopoeia, the medicinal Codonopsis radix includes Codonopsis pilosula (Franch.) Nannf., Codonopsis pilosula Nannf. var. modesta (Nannf.) L. T. Shen and Codonopsis tangshen Oliv.), Poria cocos (Schw.) Wolf [15g; Polyporaceae; Poria], Atractylodes macrocephala Koidz. [15g; Asteraceae; Atractylodis macrocephalae rhizoma], Epimedium sp. [10g; Berberidaceae; Epimedii folium] (The specific species was not specified in the original study.According to the Chinese Pharmacopoeia, the medicinal Epimedii folium includes Epimedium brevicornu Maxim., Epimedium sagittatum (Sieb. et Zucc.) Maxim., Epimedium pubescens Maxim. and Epimedium koreanum Nakai.), Curculigo orchioides Gaertn. [10g; Hypoxidaceae; Curculiginis Rhizoma], Cuscuta sp. [15g; Convolvulaceae; Cuscutae Semen] (The specific species was not specified in the original study.According to the Chinese Pharmacopoeia, the medicinal Cuscutae Semen includes Cuscuta chinensis Lam. and Cuscuta australis R.Br.), Taxillus chinensis (DC.) Danser [15g; Loranthaceae; Taxilli herba], Citrus reticulata Blanco [12g; Rutaceae;Citri reticulatae pericarpium], Atractylodes sp. [15g; Asteraceae; Atractylodis rhizoma] (The specific species was not specified in the original study.According to the Chinese Pharmacopoeia, the medicinal Atractylodis rhizoma includes Atractylodes lancea (Thunb.) DC. and Atractylodes chinensis (DC.) Koidz.), Nelumbo nucifera Gaertn. [Nelumbonaceae; Nelumbinis Folium], Cyperus rotundus L. [10g; Cyperaceae; Cyperi rotundi radix et rhizoma] | Letrozole + HFD induced PCOS with IR in rats | Activated the PI3K/AKT insulin signaling pathway | The specific extraction method was not specified. | (Zhang et al., 2025) |
| Kuntai  Capsule | Rehmannia glutinosa Libosch. [Scrophulariaceae; Rehmanniae radix], Coptis sp. [Ranunculaceae; Coptidis rhizoma] (The specific species was not specified in the original study.According to the Chinese Pharmacopoeia, the medicinal Coptidis rhizoma includes Coptis chinensis Franch., Coptis deltoidea C. Y. Cheng et Hsiao and Coptis teeta Wall.), Paeonia lactiflora Pall. [Ranunculaceae; Paeoniae Alba Radix], Scutellaria baicalensis Georgi [Lamiaceae; Scutellariae radix], Equus asinus Linnaeus [Equidae; Asini Corii Colla], Poria cocos (Schw.) Wolf [12g; Polyporaceae; Poria]  The specific dosages (in grams) of the traditional Chinese medicines involved in this study were not clearly indicated in the original data. | DHEA + HFD induced PCOS with IR in rats | NA | The specific extraction method was not specified. | (Huang et al., 2024a) |
| Liuwei  Dihuang  Pills | Rehmannia glutinosa Libosch. [Scrophulariaceae; Rehmanniae radix], Cornus officinalis Siebold & Zucc. [Cornaceae; Corni fructus], Dioscorea opposita Thunb. [Dioscoreaceae; Dioscoreae oppositae rhizoma], Alisma orientale (Sam.) Juzep. [Alismataceae; Alismatis rhizoma], Paeonia suffruticosa Andr. [Ranunculaceae; Paeoniae suffruticosae cortex], Poria cocos (Schw.) Wolf [ Polyporaceae; Poria]  The specific dosages (in grams) of the traditional Chinese medicines involved in this study were not clearly indicated in the original data. | Letrozole + HFD induced PCOS with IR in rats | Activated the PI3K/AKT insulin signaling pathway | The specific extraction method was not specified. | (Qiu et al., 2020) |
| Modified  Cangfu  daotan  Decoction | Atractylodes lancea (Thunb.) DC. [Asteraceae; Atractylodis Rhizoma], Cyperus rotundus L. [10g; Cyperaceae;Cyperi rhizoma], Pinellia ternata (Thunb.) Makino [10g; Araceae;Pinelliae rhizome], Citrus reticulata Blanco [6g; Rutaceae;Citri reticulatae pericarpium], Poria cocos (Schw.) Wolf [12g; Polyporaceae; Poria], Astragalus membranaceus (Fisch.) Bge. [30g; Fabaceae; Astragali radix] (The specific variety was not specified in the original study.According to the Chinese Pharmacopoeia, the medicinal Astragali radix includes Astragalus membranaceus (Fisch.) Bge. var. mongholicus (Bge.) Hsiao and Astragalus membranaceus (Fisch.) Bge.)], Acorus tatarinowii Schott [10g; Araceae; Acori tatarinowii rhizoma], Dioscorea opposita Thunb. [20g; Dioscoreaceae; Dioscoreae oppositae rhizoma], Angelica sinensis (Oliv.) Diels [10g; Apiaceae; Angelicae sinensis radix], Salvia miltiorrhiza Bunge [15g; Lamiaceae;Salviae miltiorrhizae radix et rhizoma], Epimedium brevicornu Maxim. [15g; Berberidaceae; Epimedii folium], Gleditsia sinensis Lam. [10g; Fabaceae; Gleditsiae spina] | Letrozole +HFD induced PCOS with IR in rats | Inhibited inflammation、enhanced insulin signaling transduction | The specific extraction method was not specified. | (Liu et al., 2022) |
| Modified  Banxia  Xiexin  Decoction | Pinellia ternata (Thunb.) Makino [9g; Araceae;Pinelliae rhizoma], Scutellaria baicalensis Georgi [20g; Lamiaceae; Scutellariae radix], Coptis sp. [10g; Ranunculaceae; Coptidis rhizoma] (The specific species was not specified in the original study.According to the Chinese Pharmacopoeia, the medicinal Coptidis rhizoma includes Coptis chinensis Franch., Coptis deltoidea C. Y. Cheng et Hsiao and Coptis teeta Wall.), Zingiber officinale Rosc. [9g; Zingiberaceae; Zingiberis rhizoma], Codonopsis sp. [12g; Campanulaceae; Codonopsis radix] (The specific species was not specified in the original study.According to the Chinese Pharmacopoeia, the medicinal Codonopsis radix includes Codonopsis pilosula (Franch.) Nannf., Codonopsis pilosula Nannf. var. modesta (Nannf.) L. T. Shen and Codonopsis tangshen Oliv.), Glycyrrhiza sp. [12g; Fabaceae; Glycyrrhizae radix et rhizoma] (The specific species was not specified in the original study.According to the Chinese Pharmacopoeia, the medicinal Glycyrrhizae radix et rhizoma includes Glycyrrhiza uralensis Fisch., Glycyrrhiza glabra L. and Glycyrrhiza inflata Bat.)], Ziziphus jujuba Mill. [9g; Rhamnaceae; Ziziphi jujubae fructus], Epimedium sp. [15g; Berberidaceae; Epimedii folium] (The specific species was not specified in the original study.According to the Chinese Pharmacopoeia, the medicinal Epimedii folium includes Epimedium brevicornu Maxim., Epimedium sagittatum (Sieb. et Zucc.) Maxim., Epimedium pubescens Maxim. and Epimedium koreanum Nakai.), Lycium barbarum L. [30g; Solanaceae; Lycii Barbari Fructus] | LETROZOLE+HFD induced PCOS with IR in rats | Reshaped the structural composition of the intestinal microbiota | The specific extraction method was not specified. | (Zhao et al., 2022) |
| Qigong  Wan | Pinellia ternata (Thunb.) Makino [15g; Araceae;Pinelliae rhizoma], Citrus reticulata Blanco [3g; Rutaceae; Pericarpium Citri Reticulatae Externum], Poria cocos (Schw.) Wolf [15g; Polyporaceae; Poria], Atractylodes macrocephala Koidz. [30g; Asteraceae; Atractylodis macrocephalae rhizoma], Cyperus rotundus L. [30g; Cyperaceae; Cyperi rhizoma], Ligusticum chuanxiong Hort. [30g; Apiaceae; Chuanxiong Rhizoma], Massa Medicata Fermentata [15g; Fermented processed product (non-plant family); Massa Medicata Fermentata], Glycyrrhiza uralensis Fisch. ex-DC [3g; Fabaceae; Glycyrrhizae radix et rhizoma] | Letrozole + HFD induced PCOS with IR in mice | Inhibited inflammation | Heated at 100 °C for 1 hour, performed twice. After the extract was filtered through cheesecloth, it was subjected to vacuum filtration and concentration using a rotary evaporator. Finally, the resulting solution was freeze-dried into a powder at -80 °C. Before use, the powder was redissolved in distilled water, diluted to an appropriate concentration, and then filtered through a 0.22 μm microporous membrane. | (Zheng et al., 2023) |
| Shengui  yangrong  Decoction | Platycladus orientalis (Linn.) Franco [10g; Cupressaceae; Platycladi Semen], Polygala tenuifolia Willd. [9g; Polygalaceae; Polygalae Radix], Lycium barbarum L. [9g; Solanaceae; Lycii Barbari Fructus], Acorus tatarinowii Schott [15g; Araceae; Acori tatarinowii rhizoma], Poria cocos (Schw.) Wolf [9g; Polyporaceae; Poria], Salvia miltiorrhiza Bunge [12g; Lamiaceae;Salviae miltiorrhizae radix et rhizoma], Angelica sinensis (Oliv.) Diels [10g; Apiaceae; Angelicae sinensis radix], Dioscorea opposita Thunb. [15g;Dioscoreaceae; Dioscoreae oppositae rhizoma] , Ligusticum chuanxiong Hort. [6g; Apiaceae; Chuanxiong Rhizoma] | DHEA+HFD induced PCOS with IR in rats | Inhabited of the EGFR/PI3K/AKT pathway | The specific extraction method was not specified. | (An et al., 2025) |
| Woxuan  Zhongzhou  Formula | Atractylodes macrocephala Koidz. [30g; Asteraceae; Atractylodis macrocephalae rhizoma], Astragalus membranaceus [15g; Fabaceae; Astragali radix] (The specific species was not specified in the original study. According to the Chinese Pharmacopoeia, the medicinal Astragali radix includes two species: Astragalus membranaceus (Fisch.) Bge.var.mongholicus(Bge.) Hsiao and Astragalus membranaceus (Fisch.) Bge.), Citrus reticulata Blanco [15g; Rutaceae;Citri reticulatae pericarpium], Astragalus membranaceus [15g; Fabaceae; Astragali radix] (The specific species was not specified in the original study. According to the Chinese Pharmacopoeia, the medicinal Astragali radix includes two species: Astragalus membranaceus (Fisch.) Bge.var.mongholicus(Bge.) Hsiao and Astragalus membranaceus (Fisch.) Bge.), Cimicifuga foetida L. [10g; Ranunculaceae; Cimicifugae rhizoma], Bupleurum chinense DC. [10g; Apiaceae; Bupleuri radix], Angelica sinensis (Oliv.) Diels[9g;Apiaceae; Angelicae sinensis radix] , Glycyrrhiza uralensis Fisch [6g;Fabaceae; Glycyrrhizae radix et rhizoma] , Poria cocos (Schw.) Wolf [10g;Polyporaceae; Poria] , Taxillus chinensis (DC.) Danser [15g; Loranthaceae; Taxilli herba], Cyperus rotundus L. [10g; Cyperaceae; Cyperi rhizoma], Achyranthes bidentata Blume [10g; Amaranthaceae; Achyranthis Bidentatae Radix], Lycium barbarum L. [10g; Solanaceae; Lycii Barbari Fructus], Morinda officinalis How [10g; Rubiaceae; Morindae officinalis radix], Pueraria lobata (Willd.) Ohwi [15g; Fabaceae; Puerariae lobatae radix] | DHEA+HFD induced PCOS with IR in mice | By activated AMPK/PGC1-α pathway | The WXZZ decoction was dissolved in heated (60 °C) deionized water to obtain a 3.0 g/mL stock solution and stored at 4 °C. | (Liu et al., 2025) |
| Yulin  Mixture | Epimedium sp. [15g; Berberidaceae; Epimedii folium] (The specific species was not specified in the original study.According to the Chinese Pharmacopoeia, the medicinal Epimedii folium includes Epimedium brevicornu Maxim., Epimedium sagittatum (Sieb. et Zucc.) Maxim., Epimedium pubescens Maxim. and Epimedium koreanum Nakai.), Polygonatum sp. [12g; Asparagaceae; Polygonati rhizoma] (the specific species was not specified in the original study; according to the Chinese Pharmacopoeia, the medicinal Polygonati rhizoma includes Polygonatum sibiricum Redouté, Polygonatum kingianum Coll. et Hemsl. and Polygonatum cyrtonema Hua.), Dipsacus asperoides C. Y. Cheng et T. M. Ai. [12g; Dipsacaceae; RADIX DIPSACI], Cyperus rotundus L.9g; Cyperaceae;Cyperi rhizoma], Acorus tatarinowii Schott [9g; Araceae; Acori tatarinowii rhizoma], Glycyrrhiza sp. [6g; Fabaceae; Glycyrrhizae radix et rhizoma] (The specific species was not specified in the original study.According to the Chinese Pharmacopoeia, the medicinal Glycyrrhizae radix et rhizoma includes Glycyrrhiza uralensis Fisch., Glycyrrhiza glabra L. and Glycyrrhiza inflata Bat.) | DHEA-induced PCOS with IR in mice | Androgen levels were reduced | First, all six Chinese herbs in the formula were boiled in 500 ml of water for 20 minutes. The liquid was then filtered, and another 500 ml of water was added to the residual herbs, which were boiled again for 20 minutes. After that, the two extracts were combined and concentrated to a liquid containing 0.6 g of crude drug per milliliter. Finally, the water extract was further purified by centrifugation at 10,000 revolutions per minute for 15 minutes. | (Zhang and Xu, 2021)  （Preliminary study of Yulin mixture affecting the miR-320/SF-1/Cyp19a1 on mouse polycystic ovary syndrome model） |
| Yulin  Tongbu  Formula | Codonopsis pilosula (Franch.) Nannf. [45g; Campanulaceae; Codonopsis Wall. radix]; Astragalus mongholicus Bunge [30g; Fabaceae; Astragali radix]; Atractylodes macrocephala Koidz. [30g; Asteraceae; Atractylodis macrocephalae rhizoma]; Actaea cimicifuga L. [10g; Ranunculaceae; Cimicifugae rhizoma]; Citrus reticulata Blanco [10g; Rutaceae;Citri reticulatae pericarpium]; Poria cocos (Schw.) Wolf [15g; Polyporaceae; Poria]; Pinellia ternata (Thunb.) Makino [10g; Araceae;Pinelliae rhizoma]; Atractylodes lancea (Thunb.) DC. [30g; Asteraceae; Atractylodis lanceae rhizoma]; Spatholobus suberectus Dunn [30g; Fabaceae; Spatholobi Caulis]; Cyperus rotundus L. [10g; Cyperaceae; Cyperi rotundi radix et rhizoma]; Cuscuta chinensis Lam. [20g; Convolvulaceae; Cuscutae Semen]; Morinda officinalis How [15g; Rubiaceae; Morindae officinalis radix] | DHEA + HFD-induced PCOS with IR in mice | Remodeled the gut microbiota composition | First, the herbs were soaked in distilled water at room temperature for 1 hour to soften the raw materials and facilitate the extraction of water-soluble components in subsequent steps. After the herbs were boiled for 2 hours, they were filtered through filter paper. The above boiling and filtration process was repeated twice. Finally, the decoction was concentrated at 100°C to obtain the YLTB extract containing 2.55 g of raw herbs per mL. | (Su et al., 2023) |
| Zishen  Qingre  Lishi  Huayu  Recipe | Cornus officinalis Siebold & Zucc. [10g; Cornaceae; Corni fructus], Phellodendron chinense C.K.Schneid. [10g; Rutaceae; Phellodendri chinensis cortex], Scrophularia ningpoensis Hemsl. [10g; Scrophulariaceae; Scrophulariae radix], Sinapi’s alba L. [10g; Brassicaceae; Sinapis albae semen], Coix lacryma-jobi L. [15g; Gramineae; Coicis semen], Salvia miltiorrhiza Bunge [10g; Lamiaceae; Salviae miltiorrhizae radix et rhizoma], Prunus persica (L.) Batsch [10g; Rosaceae; Persicae semen], Glycyrrhiza uralensis Fisch. [6g; Leguminous; Glycyrrhizae radix et rhizoma], Anemarrhena asphodeloides Bunge [10g; Asparagaceae; Anemarrhenae rhizoma] | DHEA+HFD induced PCOS with IR in rats | Inhibited inflammation | The specific extraction method was not specified. | (Li et al., 2024b) |

Table 1 Animal studies on the improvement of IR in PCOS by the sole application of TCM botanical drug formulas.

The Chinese pinyin, Latin names, generic names, and Latin scientific names of the above traditional Chinese medicines are from the Chinese Pharmacopoeia (2020 Edition), Kew Plants of the World Online, and Kew Medicinal Plant Names Services (MPNS). The website of the Chinese Pharmacopoeia: https://ydz.chp.org.cn/#/, the website of Kew Plants of the World Online: https://powo.science.kew.org, and the website of Kew Medicinal Plant Names Services (MPNS): <https://www.kew.org/science/our-science/science-services/medicinal-plant-names-services>

References

An, J., Lin, T., Guo, X., Cao, Z., and Lu, Q. (2025). Regulation of the EGFR/PI3K/AKT signaling cascade using the Shengui Yangrong Decoction improves ovulation dysfunction and insulin resistance in polycystic ovary syndrome. *Fitoterapia* 182, 106407. doi: 10.1016/j.fitote.2025.106407

Guan, H.-R., Li, B., Zhang, Z.-H., Wu, H.-S., Wang, N., Chen, X.-F., et al. (2024). Exploring the efficacy and mechanism of Bailing capsule to improve polycystic ovary syndrome in mice based on intestinal-derived LPS-TLR4 pathway. *J Ethnopharmacol* 331, 118274. doi: 10.1016/j.jep.2024.118274

Huang, J.-G., Zhang, J., Xiang, R.-J., Wu, Y.-Y., He, T.-Y., Zhao, M.-N., et al. (2024a). [Mechanism of Kuntai Capsules in treatment of polycystic ovary syndrome rat models based on AGE-RAGE signal pathway]. *Zhongguo Zhong Yao Za Zhi* 49, 1082–1090. doi: 10.19540/j.cnki.cjcmm.20231115.501

Huang, Q., Li, Y., Chen, Z., Ou, H., Tan, Y., and Lin, H. (2024b). Bushenhuoluo Decoction improves polycystic ovary syndrome by regulating exosomal miR-30a-5p/ SOCS3/mTOR/NLRP3 signaling-mediated autophagy and pyroptosis. *J Ovarian Res* 17, 29. doi: 10.1186/s13048-024-01355-x

Jiang, X.-L., Tai, H., Xiao, X.-S., Zhang, S.-Y., Cui, S.-C., Qi, S.-B., et al. (2022). Cangfudaotan decoction inhibits mitochondria-dependent apoptosis of granulosa cells in rats with polycystic ovarian syndrome. *Front Endocrinol (Lausanne)* 13, 962154. doi: 10.3389/fendo.2022.962154

Li, J., Liu, D., Zhao, H., Zhang, P., Cai, F., Li, H., et al. (2024a). Chinese medicine compound prescription HeQi San ameliorates chronic inflammatory states and modulates gut flora in dehydroepiandrosterone-induced polycystic ovary syndrome mouse model. *Int Immunopharmacol* 137, 112491. doi: 10.1016/j.intimp.2024.112491

Li, X., Yi, Y., Ren, Y., Zhang, Y., Wang, C. C., Liu, C., et al. (2024b). Zishen Qingre Lishi Huayu Recipe May Ameliorate the Symptoms of PCOS Model Rats via Alleviating Systemic and Ovarian Inflammation. *Am J Reprod Immunol* 92, e13918. doi: 10.1111/aji.13918

Lian, Y., Zhao, F., and Wang, W. (2020). Use of Bao Gui capsule in treatment of a polycystic ovary syndrome rat model. *Mol Med Rep* 21, 1461–1470. doi: 10.3892/mmr.2020.10953

Liu, H., Wang, G., Sui, C., Guo, Y., and He, X. (2025). Woxuanzhongzhou formula improves DHEAS and high-fat diet-induced IR and anovulatory mice via AMPK/PGC1- α/Irisin pathway. *J Ovarian Res* 18, 7. doi: 10.1186/s13048-025-01587-5

Liu, M., Zhu, H., Zhu, Y., and Hu, X. (2021). Guizhi Fuling Wan reduces autophagy of granulosa cell in rats with polycystic ovary syndrome via restoring the PI3K/AKT/mTOR signaling pathway. *J Ethnopharmacol* 270, 113821. doi: 10.1016/j.jep.2021.113821

Liu, S., Zhang, Y., Yang, F., Gu, J., Zhang, R., Kuang, Y., et al. (2022). Modified Cangfu Daotan decoction ameliorates polycystic ovary syndrome with insulin resistance via NF-κB/LCN-2 signaling pathway in inflammatory microenvironment. *Front Endocrinol (Lausanne)* 13, 975724. doi: 10.3389/fendo.2022.975724

Qiu, Z., Dong, J., Xue, C., Li, X., Liu, K., Liu, B., et al. (2020). Liuwei Dihuang Pills alleviate the polycystic ovary syndrome with improved insulin sensitivity through PI3K/Akt signaling pathway. *J Ethnopharmacol* 250, 111965. doi: 10.1016/j.jep.2019.111965

Su, Y.-N., Wang, M.-J., Yang, J.-P., Wu, X.-L., Xia, M., Bao, M.-H., et al. (2023). Effects of Yulin Tong Bu formula on modulating gut microbiota and fecal metabolite interactions in mice with polycystic ovary syndrome. *Front Endocrinol (Lausanne)* 14, 1122709. doi: 10.3389/fendo.2023.1122709

Wang, C., Ding, C., Hua, Z., Chen, C., and Yu, J. (2020). Cangfudaotan Decoction Alleviates Insulin Resistance and Improves Follicular Development in Rats with Polycystic Ovary Syndrome via IGF-1-PI3K/Akt-Bax/Bcl-2 Pathway. *Mediators Inflamm* 2020, 8865647. doi: 10.1155/2020/8865647

Wu, Y.-Y., Li, S.-Y., Zhu, H.-Q., Zhuang, Z.-M., Shao, M., Chen, F.-L., et al. (2022). Network pharmacology integrated with experimental validation reveals the regulatory mechanism of action of Hehuan Yin decoction in polycystic ovary syndrome with insulin resistance. *J Ethnopharmacol* 289, 115057. doi: 10.1016/j.jep.2022.115057

Xu, Y., Tang, J., Guo, Q., Xu, Y., Yan, K., Wu, L., et al. (2021). Traditional Chinese Medicine formula FTZ protects against polycystic ovary syndrome through modulating adiponectin-mediated fat-ovary crosstalk in mice. *J Ethnopharmacol* 268, 113587. doi: 10.1016/j.jep.2020.113587

Ye, Y., Zhou, W., Ren, Y., Lu, J., Chen, A., Jin, R., et al. (2023). The ameliorating effects of Guizhi Fuling Wan combined with rosiglitazone in a rat ovarian model of polycystic ovary syndrome by the PI3K/AKT/NF-κB and Nrf2/HO-1 pathways. *Gynecol Endocrinol* 39, 2254848. doi: 10.1080/09513590.2023.2254848

Zhang, Q., Ren, J., Wang, F., Li, M., Pan, M., Zhang, H., et al. (2023). Chinese herbal medicine alleviates the pathogenesis of polycystic ovary syndrome by improving oxidative stress and glucose metabolism via mitochondrial Sirtuin 3 signaling. *Phytomedicine* 109, 154556. doi: 10.1016/j.phymed.2022.154556

Zhang, X., Ji, D., Zhang, Y., Du, C., Liang, L., Ahmad, A., et al. (2025). Study on the mechanism of action of berberine combined with Jianpi Yishen Huazhuo formulation in treating obese polycystic ovary syndrome by activating PI3K/AKT signaling pathway. *Gynecol Endocrinol* 41, 2462068. doi: 10.1080/09513590.2025.2462068

Zhang, Y., and Xu, L. (2021). Preliminary study of Yulin mixture affecting the miR-320/SF-1/Cyp19a1 on mouse polycystic ovary syndrome model. *Gynecol Endocrinol* 37, 546–553. doi: 10.1080/09513590.2020.1843623

Zhao, H., Chen, R., Zheng, D., Xiong, F., Jia, F., Liu, J., et al. (2022). Modified Banxia Xiexin Decoction Ameliorates Polycystic Ovarian Syndrome With Insulin Resistance by Regulating Intestinal Microbiota. *Front Cell Infect Microbiol* 12, 854796. doi: 10.3389/fcimb.2022.854796

Zheng, R., Shen, H., Li, J., Zhao, J., Lu, L., Hu, M., et al. (2023). Qi Gong Wan ameliorates adipocyte hypertrophy and inflammation in adipose tissue in a PCOS mouse model through the Nrf2/HO-1/Cyp1b1 pathway: Integrating network pharmacology and experimental validation in vivo. *J Ethnopharmacol* 301, 115824. doi: 10.1016/j.jep.2022.115824
